# Inhibiting MDM2 enhances RIPK3-mediated necroptosis and synergizes with immune checkpoint blockade therapy

**DOI:** 10.1016/j.isci.2026.115724

**Published:** 2026-04-11

**Authors:** Yingxin Wu, Hanyang Yu, Zongxu Zhang, Weihang Xiong, Zexian Zeng, Weili Liu, Hailin Tu, Xin Lin

**Affiliations:** 1 School of Basic Medical Sciences, Tsinghua Medicine, Tsinghua University, Beijing, China; 2 Tsinghua-Peking Joint Center for Life Sciences, Beijing, China; 3 Center for Quantitative Biology, Academy for Advanced Interdisciplinary Studies, Peking University, Beijing, China; 4 Changping Laboratory, Beijing, China

**Keywords:** immune response, cell biology, cancer

## Abstract

Necroptosis is a form of programmed cell death that promotes tumor immunogenicity. To identify druggable regulators of necroptosis, we performed a small-molecule inhibitor screen and identified mouse double minute 2 (MDM2) as a suppressor of tumor necrosis factor α (TNF-α)-induced necroptosis. Genetic deletion or pharmacologic inhibition of MDM2 markedly enhanced necroptosis in a receptor-interacting protein kinase 1 (RIPK1)-dependent and p53-independent manner. Mechanistically, MDM2 interacted with RIPK3 and promoted its proteasome-mediated degradation, thereby limiting RIPK3 abundance and restraining pathway activation. *In vivo*, MDM2 deficiency increased tumor cell necroptosis, promoted inflammatory remodeling of the tumor microenvironment (TME), and enhanced CD8^+^ T cell infiltration, leading to improved tumor control. In immunologically “cold” tumor models, combining MDM2 inhibition with anti-PD-1 blockade converted tumors to a T cell-inflamed state and significantly improved therapeutic efficacy, even in p53-deficient settings. These findings identify MDM2 as a regulator of TNF-α-induced necroptosis and highlight its potential as a therapeutic target for cancer immunotherapy.

## Highlights

• MDM2 deficiency promotes RIPK1-dependent necroptosis independent of p53
• MDM2 interacts with RIPK3 and promotes its degradation
• MDM2 deficiency in E.G7 cells increases TME inflammation and CD8^+^ T cell activity
• MDM2 deficiency and inhibition turn cold tumors hot and enhance anti-PD-1 efficacy

## Graphical abstract

**Figure:**
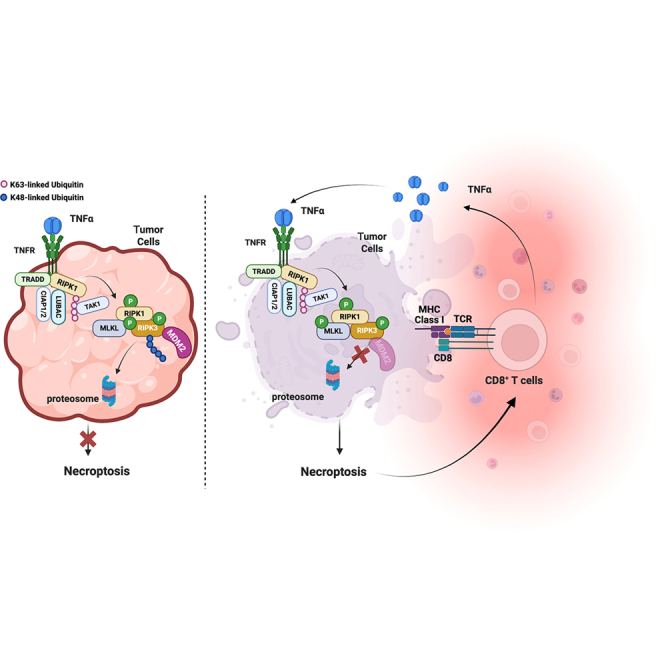

## Introduction

Necroptosis, a form of regulated cell death, has been implicated in multiple human diseases and is increasingly being recognized as a promising therapeutic target in cancers.^1^ Different from apoptosis, which forms apoptotic bodies that topologically maintain membrane integrity, necroptosis is characterized by rapid membrane permeabilization and the release of intracellular components, leading to the exposure of damage-associated molecular patterns (DAMPs), which alert immune cells of danger and trigger strong inflammatory responses.^2^^,^^3^ In tumors, enhanced necroptosis elicits a stronger inflammatory response within the tumor microenvironment (TME), promoting the activation of multiple immune cells, whereas apoptosis is generally considered immunologically silent.^4^^,^^5^ Therefore, beyond the direct cytotoxic effect of necroptosis, it generates secondary mediators that modulate immune cell activity within the TME. Elucidating the regulatory mechanisms of necroptosis may uncover new opportunities for targeting immune-related diseases, including cancers.

Among the various cellular stimuli that induce necroptosis, the tumor necrosis factor α (TNF-α) signaling pathway is one of the most extensively studied.^6^ In TNF-α-stimulated cells, receptor-interacting protein kinase 1 (RIPK1) acts as the key regulator, being either a scaffold protein leading to NF-κB activation or a kinase triggering programmed cell death.^7^ Inhibition of kinases or E3 ligases involved in the assembly of complex I, such as TAK1 or cIAP, induces RIPK1 to adopt a kinase-active conformation and undergo autophosphorylation.^8^ Thereafter, with caspase inhibition, RIPK1 interacts with RIPK3 through RIP homotypic interaction motif (RHIM) and promotes its auto-phosphorylation, which further phosphorylates mixed-lineage kinase domain-like protein (MLKL) that executes necroptosis.^9^^,^^10^ Although numerous pharmacological agents targeting RIPK1, RIPK3, and MLKL are currently under development to inhibit necroptosis,^11^ precise modulation or enhancement of necroptosis requires a more comprehensive understanding of how necroptosis is finely regulated.

Nowadays, studies have been presenting increasing evidence for the crucial role of ubiquitination in modulating necroptosis, especially the post-translational modification of RIPK1, RIPK3, and MLKL by various ubiquitin ligases and deubiquitinating enzymes (DUBs), including CHIP^12^ and A20,^13^^,^^14^ which have already been shown to regulate the stability, activity, or degradation of the necrosome components RIPK1 and RIPK3. Although substantial progress has been made in elucidating the role of ubiquitination in necroptosis, many ubiquitination events and their associated regulators have yet to be characterized, underscoring the necessity of identifying additional mechanisms governing necroptotic regulation.

In our study, to explore the potential role of ubiquitination in the regulation of necroptosis, we performed a screen using a ubiquitination-related small-molecule compound library, in which we identified mouse double minute 2 (MDM2) inhibitor as a potent enhancer of TNF-α/BV6/zVAD (T/B/Z)-induced necroptosis. MDM2 is a critical oncogene that promotes cell growth, survival, invasion, and therapeutic resistance.^15^ As an E3 ubiquitin ligase, MDM2 is most well known as the repressor of p53, the famous tumor suppressor, mediating its K48-ubiquitination and proteasomal degradation.^16^^,^^17^ Beyond its canonical function in p53 regulation, increasing evidence suggests that MDM2 also exerts multiple p53-independent functions, including promoting cell cycle progression by targeting the retinoblastoma protein (Rb) for ubiquitin-mediated degradation,^18^ and regulating ferroptosis through PPARα-mediated lipid remodeling,^19^ which collectively highlight the functional diversity of MDM2. In our study, we confirmed MDM2 as a negative regulator in TNF-α-induced necroptosis through pharmacological inhibition and genetic ablation, mechanistically verified the interaction between MDM2 and RIPK3, and linked this regulatory axis to antitumor immunotherapy, highlighting MDM2 as an important inflammation checkpoint.

## Results

### Compound screening identifies MDM2 inhibition as an enhancer of TNF-α-induced necroptosis

To identify ubiquitin-mediated regulation of necroptosis, we established a cell-based screening assay using L929 murine fibroblasts, a classic model for cell death studies, where necroptosis is induced by treatment with TNF-α, BV6 (a cIAP1/XIAP inhibitor), and zVAD-fmk (a pan-caspase inhibitor)—a standard combination that robustly triggers necroptosis.^20^ To probe the role of ubiquitination in TNF-α-induced necroptosis, we screened a focused compound library comprising 211 small molecules targeting key components of the ubiquitin system, including proteasomes, DUBs, E3 ligases, E1 and E2 enzymes, SUMOylation factors, as well as ubiquitination-related signaling pathways such as mTOR, NF-κB, and Wnt/β-catenin.

**Figure 1:**
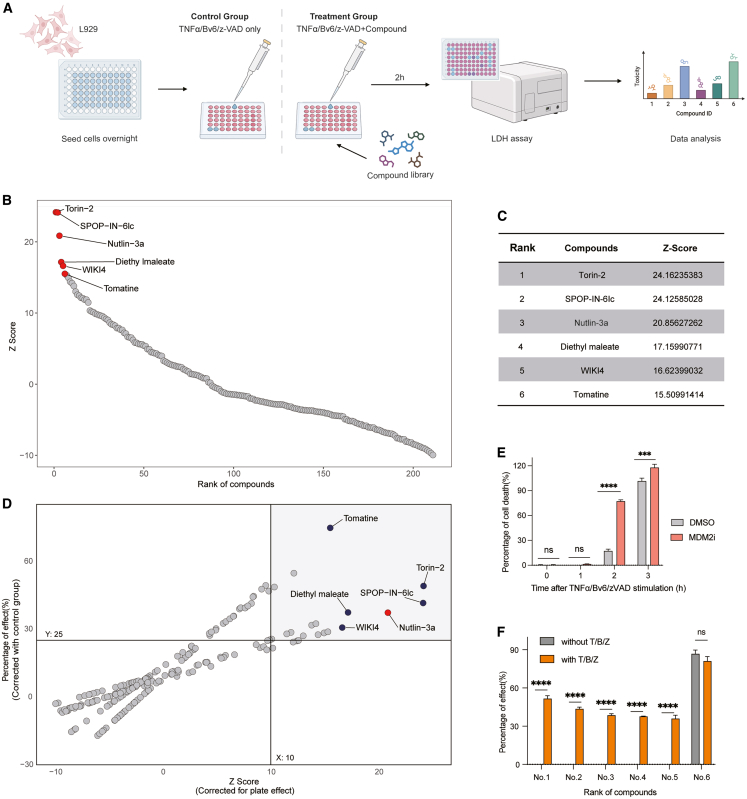
Compound library screening identifies MDM2 inhibitor as potent enhancer of TNF-α-induced necroptosis (A) Schematics of the compound library screening workflow. L929 cells were seeded overnight and treated with compounds from a ubiquitination-related chemical library and TNF-α (T), BV6 (B), and zVAD (Z) for 2 h. Cell death was assessed by LDH release assay, and data were normalized for analysis. (B) Rank plot showing Z scores of all tested compounds. The top six hits with the highest Z scores are highlighted in red, including Torin-2, SPOP-IN-6lc, Nutlin-3a, diethyl maleate, WIKI4, and tomatine. (C) Table summarizing the top six compounds ranked by *Z* score from the screening. (D) Scatterplot comparing Z scores (*x* axis) and percent enhancement of cell death (*y* axis) for all compounds after correction for plate effects. The top six hits are highlighted. (E) Time-course analysis of T/B/Z-induced cell death in the presence of DMSO or the MDM2 inhibitor Nutlin-3a. (F) Validation of the top six compounds in the presence or absence of T/B/Z stimulation in L929 cells. Cell death was measured by LDH release after 2 h. Data are represented as the mean ± SEM (standard error of the mean). *p* values were determined by two-way ANOVA with Tukey’s correction for multiple comparisons. ∗∗∗∗, *p* < 0.0001; ns, not significant. Dosages: TNF-α, 10 ng/mL; BV6, 2.5 μM; zVAD, 20 μM; compounds from the library, 10 μM.

The extent of necroptosis was quantified using a lactate dehydrogenase (LDH) release assay after 2 h of stimulation (Figure 1A). Z scores were calculated for all compounds and ranked to minimize plate-to-plate variation. The highest *Z* score in the control groups was 1.01, with 81 compounds (38% of the library) exceeding this threshold, suggesting a broad involvement of ubiquitination in necroptosis regulation. Among the top enhancers of TNF-α-induced necroptosis, Torin-2 and SPOP-IN-6IC ranked first and second, respectively (Figures 1B and 1C), targeting mammalian target of rapamycin (mTOR)^21^ and speckle-type POZ protein (SPOP), respectively,^22^ both of which have been previously reported to regulate necroptosis.

Notably, Nutlin-3, an MDM2 inhibitor, ranked third in the screen and is primarily known for its E3 ligase activity toward p53 regulation and modulating apoptosis.^23^ To validate this finding, we examined both qualitative and quantitative measures of cell death, showing that MDM2 inhibition increased necroptosis by over 30% compared with controls (Figure 1D). Further, L929 cells treated with TNF-α/BV6/zVAD (T/B/Z) plus Nutlin-3a at different time points exhibited a marked increase in cell death within 2 h, coinciding with the onset of necroptosis in DMSO-treated controls (Figure 1E), indicating that Nutlin-3a potentiates necroptosis. To rule out intrinsic cytotoxicity, we tested the compounds in the absence of T/B/Z; only tomatine in the top six hits induced cell death without T/B/Z treatment (Figure 1F). Consistently, MDM2 inhibition alone did not trigger cell death, whereas co-treatment with T/B/Z markedly enhanced necroptosis (Figure 1F). Overall, the prominence of Nutlin-3a in our screen implicates MDM2 as a previously unrecognized checkpoint in TNF-α-induced necroptosis, providing new insights into ubiquitin-mediated regulation of necroptosis and broadening our understanding of MDM2.

### MDM2 inhibition and deficiency promote RIPK1-dependent necroptosis

**Figure 2:**
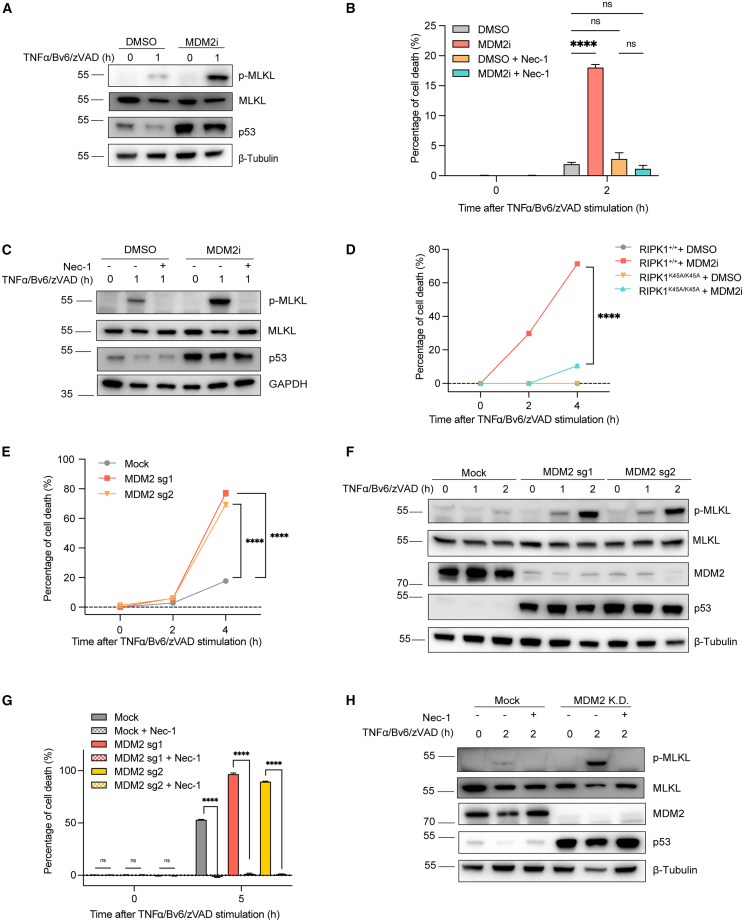
MDM2 inhibition and deficiency promote RIPK1-dependent necroptosis (A) L929 cells were treated with DMSO or Nutlin-3a followed by T/B/Z treatment for indicated time points. The cell lysates were analyzed by western blotting using indicated antibodies. (B and C) L929 cells were treated with DMSO or Nutlin-3a followed by T/B/Z treatment in the presence or absence of Nec-1 for indicated time points. (B) Cell death was determined by LDH release in the supernatant. (C) The cell lysates were analyzed by western blotting using indicated antibodies. (D) BMDMs from *Ripk1*^*+/+*^ and *Ripk1*^*K45A/K45A*^ mice were treated with DMSO or Nutlin-3a followed by T/B/Z treatment for indicated time points. Cell death was determined by LDH release in the supernatant. (E and F) MDM2 KD L929 cells were engineered by CRISPR-Cas9 technology. RFP (Mock) and MDM2 KD L929 cells were treated with T/B/Z at indicated time points. (E) Cell death was determined by LDH release in the supernatant. (F) The cell lysates were analyzed by western blotting using indicated antibodies. (G and H) Mock and MDM2 KD L929 cells were treated with T/B/Z in the presence or absence of Nec-1 at indicated time points. (G) Cell death was determined by LDH release in the supernatant. (H) The cell lysates were analyzed by western blotting using indicated antibodies. MDM2 KD refers to the MDM2 sg1 line. TNF-α, 10 ng/mL; BV6, 2.5 μM; zVAD, 20 μM; Nec-1, 10 μM. Data are represented as the mean ± SEM. *p* values were determined by ordinary two-way ANOVA with Tukey’s correction for multiple comparisons. ∗∗∗∗, *p* < 0.0001; ns, not significant.

To further validate the involvement of MDM2 in necroptosis, we examined necroptotic signaling by western blot analysis. As expected, treatment with T/B/Z in the presence of MDM2 inhibition led to increased phosphorylation of MLKL, indicating enhanced necroptosis after 1 h (Figure 2A). This effect was consistently observed in RAW264.7 cells and mouse immortalized bone marrow-derived macrophages (iBMDMs) (Figures S1A and S1B), further supporting a suppressive role for MDM2 in necroptosis induction.

To further investigate whether kinase RIPK1 is involved in necroptosis mediated by MDM2 inhibition, we treated T/B/Z-stimulated L929 cells with Nec-1, a commonly used RIPK1 kinase inhibitor, and found that Nec-1 completely rescued the enhanced necroptosis promoted by MDM2i (Figure 2B), which is in accordance with the western blotting results for phosphorylated MLKL (Figure 2C). K45A mutation was also introduced into RIPK1 in BMDMs to abolish the kinase activity of RIPK1, and, accordingly, MDM2i almost failed to trigger enhanced necroptosis in BMDM with RIPK1 K45A mutation (Figure 2D). We also excluded the impact of MDM2i on modulating NF-κB transcriptional activation by measuring the nuclear translocation of RELA (p65) upon TNF-α stimulation (Figure S1C). Collectively, these findings suggest that MDM2 inhibitor promotes RIPK1 kinase-dependent necroptosis downstream of TNFR1.

To further identify the function of MDM2, we genetically depleted MDM2 through the CRISPR-Cas9 method in L929 cells (Figure S1D) and found that MDM2 knockdown (KD) consistently enhanced T/B/Z-induced cell death (Figure 2E). Increased phosphorylation of MLKL was also observed after 1 and 2 h of T/B/Z stimulation in L929 cells with MDM2 deficiency (Figure 2F). Nec-1 was also employed and completely rescued the augmented cell death induced by T/B/Z in MDM2 KD L929 cells (Figures 2G and 2H). Taken together, these data demonstrate that both MDM2 deficiency and pharmacologic inhibition potentiate RIPK1-dependent necroptosis.

### MDM2 regulates necroptosis via RIPK3 degradation

MDM2 is well known to mediate the K48-linked ubiquitination of p53, thereby inhibiting apoptosis. Therefore, we further investigated whether the enhanced necroptosis in MDM2-deficient cells is associated with p53. Firstly, we systematically examined key proteins associated with p53-induced apoptosis signaling (BAX and PUMA) and found no significant activation of these markers during MDM2 knockout- or inhibitor-induced necroptosis (Figures S1E and S1F). Then, we knocked down p53 in MDM2-deficient L929 cells and found that the absence of p53 did not affect the enhanced necroptosis induced by MDM2 deficiency (Figure S1G). Similarly, inhibition of MDM2 also promoted cell death induced by T/B/Z treatment in p53-deficient L929 cells (Figure S1H). Taken together, these findings excluded the role of p53 in necroptosis enhancement caused by MDM2 loss or inhibition.

**Figure 3:**
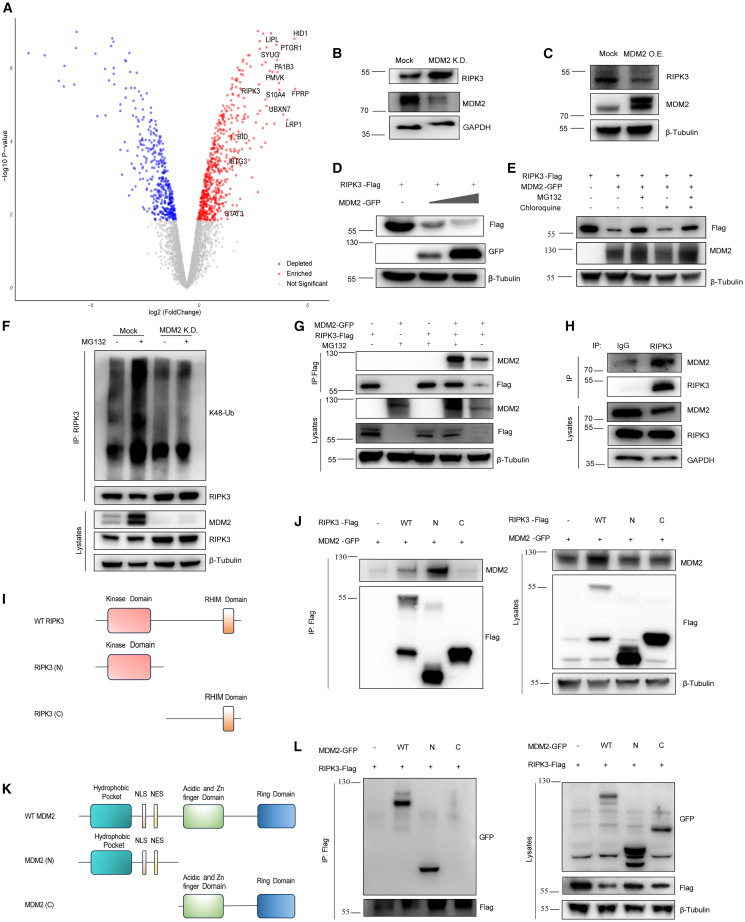
MDM2 interacts with RIPK3 and mediates the degradation of RIPK3 (A) Volcano plot showing the differential proteins between Mock and MDM2 KD L929 cells. Cutoff criteria: |log_2_ fold change| ≥ 2 and adjusted *p* value <0.05. (B) Levels of RIPK3 in Mock and MDM2 KD L929 cells, analyzed by western blotting. (C) Levels of RIPK3 in Mock and MDM2 OE (overexpression) L929 cells, analyzed by western blotting. (D) RIPK3-Flag plasmid was co-transfected with increased concentrations of MDM2-GFP in 293T cells for 24 h, then the cell lysates were analyzed by western blotting using indicated antibodies. (E) 293T cells were transfected with RIPK3-Flag and vector-GFP or MDM2-GFP in the presence of MG132 or chloroquine, then the cell lysates were analyzed by western blotting using indicated antibodies. MG132, 10 μM; chloroquine, 20 μM. (F) Endogenous coIP analysis to detect the role of MDM2 in the ubiquitination of RIPK3. Mock and MDM2 KD L929 cells were treated with or without MG132 for 4 h. MG132, 10 μM. (G) 293T cells were transfected with RIPK3-Flag and MDM2-GFP with or without MG132. Lysates were immunoprecipitated with anti-Flag beads and analyzed by western blotting using indicated antibodies. (H) Endogenous coIP analysis revealed an interaction between endogenous RIPK3 and MDM2 in L929 cells. (I) Schematic of the RIPK3 domain structure and truncation mutants used. (J) 293T cells were transfected with the indicated Flag-tagged RIPK3 and MDM2-GFP. coIP and western blotting were performed using the indicated antibodies. (K) Schematic of the MDM2 domain structure and truncation mutants used. (L) 293T cells were transfected with the indicated GFP-tagged MDM2 and RIPK3-Flag plasmids. coIP and western blotting were performed using the indicated antibodies.

To further elucidate how MDM2 deficiency sensitizes TNF-α-induced necroptosis, mass spectrometry was performed to depict the proteomic changes in MDM2-deficient cells, revealing 214 proteins that were significantly upregulated and downregulated (Table S1). Among these differential proteins, we surprisingly found that RIPK3, a member of the necrosome complex, was significantly enriched (Figure 3A). This discovery was subsequently validated through western blotting analyses, revealing RIPK3 accumulation in MDM2-deficient L929 cells (Figures 3B and S2C). Conversely, MDM2 overexpression in L929 cells led to a reduction in RIPK3 expression (Figures 3C and S2C), establishing a negative regulatory relationship between MDM2 and RIPK3. Because MDM2 is a well-known E3 ligase, we postulated that MDM2 may be involved in the degradation of RIPK3. The UbiBrowser database, encompassing E3 ligases and associated complex components, was subsequently utilized to predict proteins that potentially interact with RIPK3, among which MDM2 was identified with high confidence and ranked among the top 20 in both validated and predicted E3 ligases targeting RIPK3 (Figure S2A).

To validate the prediction above, we co-transfected RIPK3 and MDM2 in 293T cells and observed downregulation of RIPK3 in a dose-dependent manner with MDM2 overexpression (Figures 3D and S2C). However, the other RIP family member, RIPK1, was not downregulated under such conditions (Figure S2B), further demonstrating that MDM2 specifically associates with RIPK3 downregulation. The proteasome inhibitor MG132 or the lysosome inhibitor chloroquine was further used to identify the degradation pathway of RIPK3. MG132 largely rescued MDM2-induced RIPK3 degradation, whereas chloroquine had no effect (Figures 3E and S2C), suggesting that MDM2 mediates the degradation of RIPK3 via the proteasome pathway. Consistently, we confirmed that MDM2 mediates K48-linked ubiquitination of RIPK3 endogenously in L929 cells (Figures 3F and S2C). Moreover, co-immunoprecipitation (coIP) of Flag-tagged RIPK3 and GFP-tagged MDM2 demonstrated the interaction between the two proteins (Figures 3G and S2C), and this interaction was further verified endogenously in L929 cells (Figure 3H).

Regarding the interaction domains, RIPK3 mediates necroptosis through its N-terminal kinase domain and C-terminal RHIM domain (Figure 3I). Through a systematic truncation analysis, we identified that MDM2 specifically binds to RIPK3’s N-terminal kinase domain (Figure 3J). Strikingly, deletion of the C-terminal domain in RIPK3 substantially impaired MDM2-mediated degradation (Figure S2D), suggesting that the C terminus is essential for efficient ubiquitination, while the N terminus mediates physical interaction. On the other hand, domain truncation assay in MDM2 revealed that MDM2’s N-terminal region is necessary for RIPK3 binding (Figures 3K and 3L).

Overall, the above data revealed a crucial role of RIPK3 in elevated necroptosis sensitivity toward MDM2 deficiency. Concordantly, the administration of GSK’872, a RIPK3 inhibitor, completely reversed the enhanced necroptosis in MDM2-deficient L929 cells upon T/B/Z treatment (Figure S2E), further strengthening the evidence that MDM2 deficiency promotes RIPK1-dependent necroptosis by preventing the degradation of RIPK3.

### Loss of MDM2 triggers necroptosis and reshapes the inflammatory landscape in E.G7 tumors

**Figure 4:**
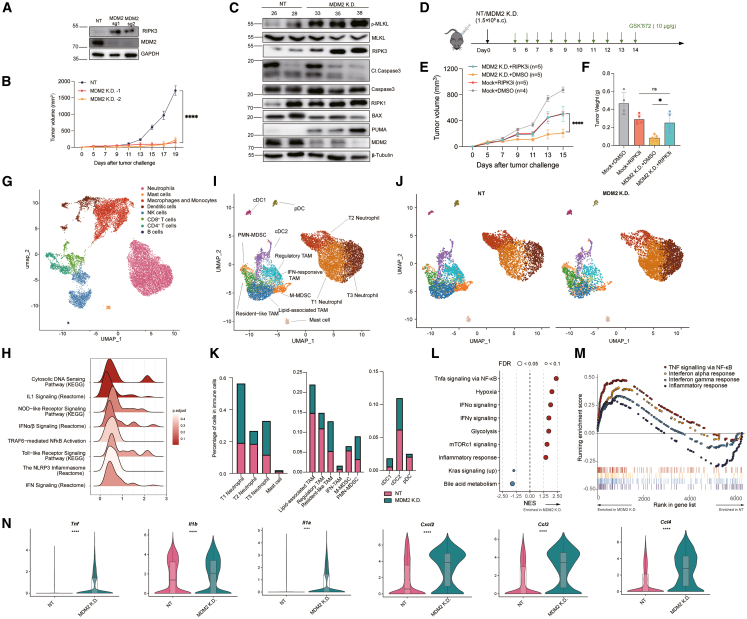
MDM2 deficiency triggers necroptosis and reshapes the inflammatory microenvironment within E.G7 tumors (A) Levels of RIPK3 in Mock and MDM2 KD E.G7-OVA cells, analyzed by western blotting. (B and C) Effect of MDM2 deficiency on E.G7-OVA tumor progression. E.G7-OVA tumors were inoculated into C57BL/6 male mice. (B) Tumor volumes were monitored (*n* = 5), and tumors were harvested on day 19, (C) Cell lysates were extracted and analyzed by western blotting using indicated antibodies. Numbers indicate individual mouse IDs. *n* represents the number of animals. (D–F) Effect of GSK’872 on MDM2-deficient E.G7-OVA tumor model. (D) Schematic of the treatment regimen. GSK’872 was administered intraperitoneally once daily from the day of tumor inoculation (day 0) through day 5 to inhibit RIPK3 kinase activity in the MDM2 KD background. (E) Line graph showing tumor volumes; tumors were harvested on day 19. (F) Bar graph showing tumor weights; *n* represents the number of animals. (G) UMAP plot showing the overall immune cell landscape from scRNA-seq of tumor-infiltrating immune cells from non-targeting (NT) and MDM2 KD E.G7-OVA tumors. Major immune cell types are color-coded. (H) Ridge plots depicting the enrichment scores of DAMP-related pathways across myeloid cell populations in NT vs. MDM2 KD tumors. (I) UMAP plot highlighting the classification of myeloid cell subpopulations, including neutrophil subsets (T1–T3), macrophage/monocyte subsets (lipid-associated TAM, regulatory TAM, resident-like TAM, IFN-responsive TAM, M-MDSCs, and PMN-MDSCs), and dendritic cell subsets (cDC1, cDC2, and pDC). (J) UMAP plots comparing the myeloid cell composition between NT and MDM2 KD tumors. (K) Bar graphs showing the relative proportions of myeloid cell subtypes between NT and MDM2 KD tumors. (L) GSEA results of differentially expressed genes in myeloid cells, showing the enrichment of inflammatory and immune-related pathways in MDM2 KD tumors. (M) GSEA enrichment plots for selected upregulated pathways in myeloid cells from MDM2 KD tumors. (N) Violin plots showing the expression of representative pro-inflammatory genes (*Tnf*, *Il1b*, *Il1a*, *Cxcl2*, *Ccl3*, and *Ccl4*) in myeloid cells from NT and MDM2 KD tumors. Data are represented as the mean ± SEM. *p* values were determined by ordinary two-way ANOVA with Tukey’s correction for multiple comparisons in (B), (E), and (F), and by ordinary one-way ANOVA in (N). ∗, *p* < 0.05, ∗∗∗∗, *p* < 0.0001, ns, not significant.

Necroptosis participates in multiple biological processes, particularly tumor immunosurveillance. While MDM2 is a well-established oncogene best known for its role in inhibiting apoptosis, our above study identified MDM2 as a negative regulator of necroptosis, which prompted us to investigate how MDM2-mediated suppression of necroptosis influences tumor progression. To this end, we employed the murine T cell lymphoma cell line E.G7, which is highly sensitive to T/B/Z-induced necroptosis *in vitro* (Figure S3A). In the E.G7 model, MDM2 deficiency markedly upregulated RIPK3 expression (Figure 4A), and both genetic ablation and pharmacologic inhibition of MDM2 markedly increased necroptosis (Figures S3B and S3C), consistent with the phenotypes observed in L929 cells. To assess whether this mechanism extends to human tumors, we examined the human colon cancer cell line SW480. Similarly, MDM2 KD in SW480 cells increased RIPK3 protein levels (Figure S4A). Both genetic depletion and pharmacological inhibition of MDM2 enhanced T/B/Z-induced necroptosis, as assessed by LDH release and western blotting (Figures S4B–S4E), indicating that MDM2-mediated suppression of necroptosis is conserved across species.

To evaluate these effects *in vivo*, we subcutaneously implanted E.G7 cells with or without MDM2 deficiency into C57BL/6 mice and monitored tumor growth. MDM2-deficient tumors exhibited delayed growth from day 13 onward (Figure 4B) and reduced tumor weight (Figures S3D and S3E). Furthermore, western blotting of tumor lysates confirmed increased MLKL phosphorylation, indicating enhanced necroptosis (Figure 4C). To determine whether the enhanced tumor control is functionally linked to necroptosis, the necroptosis inhibitor GSK’872 was intraperitoneally injected into E.G7 tumor-bearing mice with or without MDM2 deficiency (Figure 4D). Strikingly, GSK’872 administration significantly attenuated the tumor regression observed in MDM2-deficient tumors (Figures 4E, 4F, and S3F), demonstrating that necroptosis is essential for the antitumor effects associated with MDM2 loss.

Unlike apoptosis, which is typically immunologically silent, necroptosis is able to promote immune activation via the release of DAMPs. To assess the impact of MDM2 deficiency on the immune landscape, we performed single-cell RNA sequencing (scRNA-seq) of CD45^+^ immune cells isolated from E.G7 tumors with or without MDM2 deficiency. Data from both groups were aggregated, and uniform manifold approximation and projection (UMAP) analysis was applied to define consistent cell clusters (Figures 4G and S5A), which were subsequently annotated based on canonical marker genes (Figure S5B).

Because DAMPs are primarily sensed by innate immune receptors on myeloid cells,^24^ we first examined the expression profiles of DAMP-sensing receptors. Multiple pattern recognition receptors (PRRs), including members of the TLR family, P2RX7, CLEC4E, and TREM1, were found to be primarily expressed in myeloid cells, with comparable expression levels between control and MDM2 KD groups (Figure S5C). Despite the similar receptor expression, myeloid cells in the MDM2 KD group exhibited significant enrichment of DAMP-associated signaling pathways, such as cytosolic DNA sensing pathway, NOD-like receptor signaling, Toll-like receptor signaling, and inflammasome assembly (Figure 4H). Given the pivotal role of myeloid cells in DAMP sensing, we performed subclustering of myeloid cells based on published literature,^25^^,^^26^ which revealed a marked decrease in several immunosuppressive myeloid populations, including lipid-associated tumor-associated macrophages (APOE^hi^ TAMs), regulatory TAMs (MMP14^hi^ CD274^+^), and monocytic myeloid-derived suppressor cells (M-MDSCs) (Figures 4I–4K). To gain insights into the functional differences underlying the alterations in myeloid cell compositions, gene set enrichment analysis (GSEA) was performed, which uncovered that MDM2 deficiency was enriched for numerous gene sets associated with inflammatory responses, including TNF signaling via NF-κB and multiple interferon signaling pathways (Figures 4L and 4M). Consistently, Gene Ontology (GO) analysis also revealed enhanced regulation of inflammatory response and TNF superfamily cytokine production (Figure S5D). Several important pro-inflammatory mediators, including the cytokines TNF-α, IL-1α, and IL-1β, as well as the chemokines CXCL2, CCL3, and CCL4 were significantly elevated in myeloid cells from MDM2-deficient tumors (Figure 4N), further supporting an overall pro-inflammatory shift in the TME.

### MDM2 deficiency enhances necroptosis-driven CD8^+^ T cell recruitment and antitumor immunity

**Figure 5:**
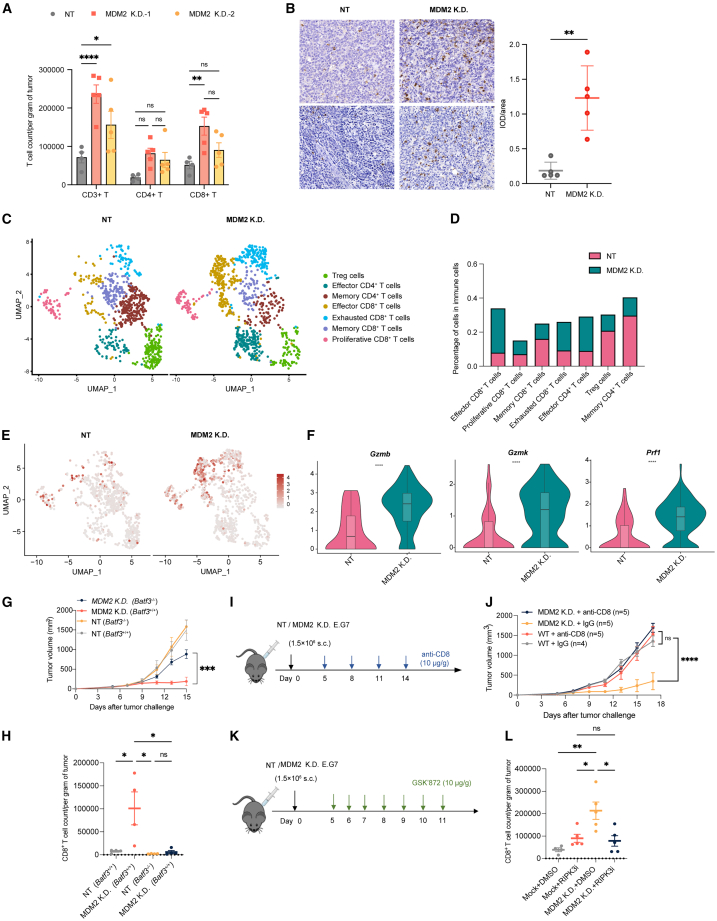
MDM2 deficiency enhances CD8^+^ T cell infiltration and cytotoxicity in the E.G7 tumor model (A) Quantification of tumor-infiltrating CD3^+^, CD4^+^, and CD8^+^ T cells in NT and MDM2 KD E.G7 tumors by flow cytometry, normalized to tumor weight. (B) Representative IHC staining for CD8^+^ T cells in NT and MDM2 KD tumors (left), and quantification of IOD/area (right). Scale bars, 50 μm. (C) UMAP visualization of T cell subclusters from scRNA-seq data of NT and MDM2 KD E.G7 tumors. (D) Proportion of each T cell subset in NT versus MDM2 KD tumors. (E) UMAP plots showing the cytotoxicity scores of T cells calculated based on cytotoxic gene signatures. (F) Violin plots showing the expression of cytotoxicity-related effector molecules (*Gzmb*, *Gzmk*, and *Prf1*) in T cells from NT and MDM2 KD tumors. (G and H) Functional assessment of DC involvement in CD8^+^ T cell infiltration, using *Batf3*^−/−^ female mice (*n* = 4), in which cDC1s are selectively absent. (G) Tumor growth curves. (H) Intratumoral CD8^+^ T cell numbers are shown. *n* represents the number of animals. (I and J) Effect of anti-CD8 on the growth of MDM2-deficient E.G7-OVA tumors. (I) Experimental design showing that anti-CD8 was injected into C57BL/6 male mice intraperitoneally once every three days, (J) with consistent tumor volume monitoring. *n* represents the number of animals. (K and L) Effect of RIPK3 kinase inhibition on CD8^+^ T cell infiltration in MDM2 KD tumors. (K) Experimental schematic of GSK’872 treatment in C57BL/6 male mice. (L) Tumors were harvested on day 11, and CD8^+^ T cells per gram of tumor were quantified by flow cytometry (*n* = 4 in Mock and DMSO groups; *n* = 5 in the other three groups). *n* represents the number of animals. Data are represented as the mean ± SEM. *p* values were determined by ordinary two-way ANOVA with Tukey’s correction for multiple comparisons in (A), (G), (H), (J), and (L) and by ordinary one-way ANOVA in (B) and (F). ∗, *p* < 0.05, ∗∗, *p* < 0.01, ∗∗∗, *p* < 0.001, ∗∗∗∗, *p* < 0.0001 ns, not significant.

T lymphocyte-mediated cytotoxicity, predominantly orchestrated by effector CD8^+^ T cells, constitutes the primary force of antitumor immunity.^27^ Since MDM2 depletion reprograms the tumor immune landscape toward a more inflammatory state, we next examined whether MDM2 deficiency in tumors promotes T cell recruitment. As expected, MDM2 KD in E.G7 tumor increased the abundance of T cells, particularly CD8^+^ T cells, which was further confirmed by immunohistochemistry (IHC) staining (Figures 5A and 5B). To further characterize the alteration in T lymphocytes and validate the increase in CD8^+^ T cells, we focused on the T lymphocyte cluster from our scRNA-seq dataset and stratified them into functional subsets based on canonical marker expression (Figure S5E). The subclustering analysis further revealed a substantial increase in effector CD8^+^ T cells in MDM2-deficient tumors (Figures 5C and 5D). Functionally, these effector CD8^+^ T cells exhibited enhanced cytotoxicity, supported by a curated cytotoxicity gene set^28^ (Figure 5E), as well as elevated expressions of granzymes and perforin (*Prf1*) (Figure 5F).

Given the marked increase in effector CD8^+^ T cells in MDM2-deficient tumors, we next explored potential upstream immune regulators that might drive this enhanced T cell response. Dendritic cells (DCs) are well known for their role in sensing DAMPs and activating CD8^+^ T cells through antigen cross-priming in the draining lymph nodes, thereby promoting T cell migration to the tumor and contributing to antitumor immunity.^29^

In our scRNA-seq datasets, GO analysis revealed enhanced antigen presentation and T cell activation capacity in DCs from MDM2-deficient tumors (Figure S6A). Specifically, we observed an increased proportion of conventional type 1 dendritic cells (cDC1), the subset primarily responsible for cross-priming CD8^+^ T cells (Figure S6B). Concordantly, *in vivo* experiments revealed a higher frequency of activated DC populations (CD80^+^ or CD86^+^) in the draining lymph nodes harvested from MDM2-deficient E.G7 tumor-bearing mice (Figures S6C and S6D). To further delineate the role of cDC1 in this process, we utilized *Batf3*-knockout mice, which lack functional cDC1. Strikingly, the loss of functional cDC1 in *Batf3*^−/−^ mice markedly impaired CD8^+^ T cell infiltration and abrogated the tumor growth suppression observed in MDM2-deficient tumors (Figures 5G, 5H, S6E, and S6F), highlighting the essential role of cDC1 in this context.

Given these findings, we next sought to determine whether CD8^+^ T cells are indispensable for the regression of MDM2-deficient tumors. While the depletion of CD8^+^ T cells had only minor effects on tumor control in NT tumors, the administration of anti-CD8 monoclonal antibodies nearly completely reversed the tumor growth inhibition in MDM2-deficient tumors (Figures 5I and 5J), underscoring the critical contribution of CD8^+^ T cells to this process. Furthermore, pharmacological inhibition of necroptosis significantly reduced CD8^+^ T cell infiltration in MDM2-deficient E.G7 tumors (Figures 5K and 5L), supporting a link between MDM2 deficiency-induced necroptosis and enhanced CD8^+^ T cell recruitment that mediates effective tumor suppression.

### MDM2 deficiency or inhibition enhances antitumor immunity and facilitates ICB treatment

**Figure 6:**
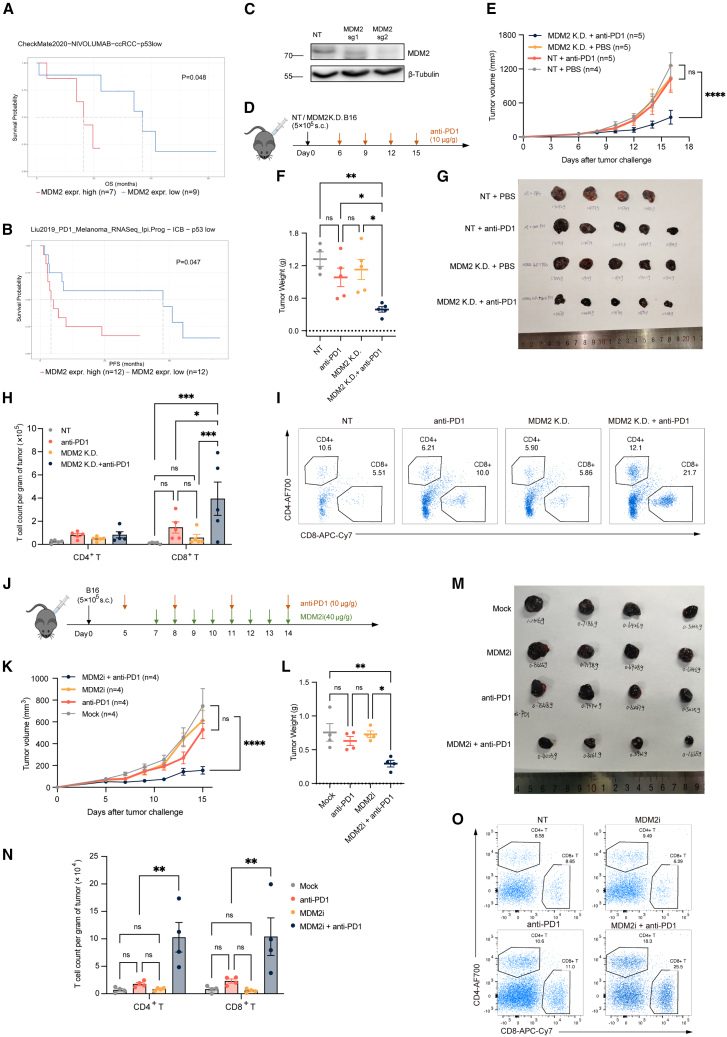
MDM2 deficiency and inhibition promote anti-PD-1 efficacy by enhancing recruitment of T cells within B16 tumors (A and B) Kaplan-Meier survival analysis depicting the difference in overall survival or progression-free survival between the group with high transcript levels of MDM2 and the group with lower transcript levels of MDM2 in patients with different types of cancers including (A) ccRCC (B) and melanoma with lower transcripts of p53. Data were obtained from clinical outcomes reported in published papers. (C) Knockout efficiency of MDM2 in B16 tumors was evaluated by western blotting with antibodies against MDM2. (D–I) Effect of MDM2 deficiency on anti-PD-1 therapy on B16 tumor model. (D) Experimental design showing that C57BL/6 male mice were inoculated subcutaneously with NT or MDM2 KD B16, followed by anti-PD-1 injection intraperitoneally once every three days. (E) Line graph showing tumor volumes. (F and G) Tumors were harvested on day 16 and then weighed. (H and I) MDM2 deficiency promoted T cell infiltration with anti-PD-1 in B16. (H) Quantification of infiltrating CD4^+^ and CD8^+^ T cell counts per gram of tumor. (I) The percentage of CD4^+^ and CD8^+^ subsets in infiltrated immune cells. *n* represents the number of animals. (J–O) Effect of the MDM2 inhibitor Nutlin-3a on anti-PD-1 therapy in B16 tumor model. (J) Experimental design showing that C57BL/6 male mice were inoculated subcutaneously with WT B16 followed by MDM2i injection every day and anti-PD-1 once every three days intraperitoneally. (K) Line graph showing tumor volumes. (L and M) Tumors were harvested on day 15 and then weighted. (N and O), MDM2 inhibitor Nutlin-3a promoted T cell infiltration with anti-PD-1 in B16 tumors. (N) Quantification of infiltrating CD4^+^ and CD8^+^ T cell counts per gram of tumor. (O) The percentage of CD4^+^ and CD8^+^ subsets in infiltrated immune cells. *n* represents the number of animals. Data are represented as the mean ± SEM. *p* values were determined by the log-rank test (A and B) and ordinary two-way ANOVA with Tukey’s correction for multiple comparisons (E, F, H, K, L, and N). ∗, *p* < 0.05; ∗∗, *p* < 0.01; ∗∗∗, *p* < 0.001; ∗∗∗∗, *p* < 0.0001; ns, not significant.

With the development of cancer immunotherapy, immune checkpoint blockade (ICB) has been widely applied and shown promising therapeutic effects across multiple cancer types. However, certain tumors remain unresponsive to ICB, often characterized by a “cold” immune environment.^30^ Notably, MDM2/MDM4 amplification was previously reported to be a prognostic factor for resistance to anti-PD-1 therapy in the Memorial Sloan Kettering Cancer Center (MSKCC) cohorts,^31^ and we further confirmed this association in additional patient cohorts with low *Tp53* expression and treated with anti-PD-1 therapy, in clear cell renal carcinoma (ccRCC)^32^ and melanoma^33^ (Figures 6A and 6B). These clinical correlations, together with our experimental evidence that MDM2 inhibits tumor necroptosis, not only provide a mechanistic explanation for the observed resistance but also suggest that targeting MDM2 could be a viable strategy to remodel the inflammatory TME and convert “cold” tumors into “hot”, thereby enhancing responsiveness to ICB.

Therefore, we employed the B16 melanoma cell line, a well-established model with a cold TME that can be induced to undergo necroptosis *in vivo*.^34^ Anti-PD-1 monoclonal antibodies were administered intraperitoneally to C57BL/6 mice bearing either wild-type (WT) or MDM2 KD B16 cells (Figures 6C and 6D). As expected, anti-PD-1 treatment exhibited minimal effects against WT B16 tumors but produced significant therapeutic effects in MDM2-deficient B16 tumors, whereas MDM2-deficient B16 tumors without ICB treatment also showed minimal growth inhibition (Figures 6E–6G). Given that MDM2 deficiency promotes necroptosis in tumor cells and a strongly immunosuppressive feature of B16 tumors, we supposed a similar inflamed TME in MDM2-deficient B16 tumors in the context of ICB treatment, thus recruiting more T cells. Indeed, the flow cytometry analysis of tumors harvested on day 16 revealed dramatic enrichment of CD8^+^ T cells in MDM2-deficient tumors treated with anti-PD-1 but not in tumors either receiving anti-PD-1 treatment or with MDM2-deficiency alone (Figures 6H and 6I), indicating that MDM2 deficiency functions as an important immune activator to greatly enhance T cell recruitment in the context of ICB treatment.

We next evaluated the therapeutic efficacy of combining pharmacologic MDM2 inhibition with anti-PD-1 therapy in the B16 model. MDM2 inhibitor was administered intraperitoneally once daily starting on day 7 after tumor implantation, and anti-PD-1 was given every three days starting on day 5 (Figure 6J). Consistent with the genetic model, single-agent treatment with either MDM2 inhibitor or anti-PD-1 showed minimal antitumor activity, whereas the combination achieved significant tumor control (Figures 6K–6M). On day 15, the analysis of tumor-infiltrating lymphocytes showed that the combination group exhibited markedly increased infiltration of both CD8^+^ and CD4^+^ T cells relative to single-agent treatments (Figures 6N and 6O), confirming that the pharmacologic MDM2 blockade can potentiate ICB efficacy in “cold” tumors.

As MDM2 inhibition promotes necroptosis independent of p53, we further tested the combination therapy in a p53-knockout B16 model (Figure S7A). Mice were implanted with p53-deficient B16 tumors and treated with MDM2 inhibitor and anti-PD-1 at the same dosages and schedules as above (Figure S7B). Addition of the MDM2 inhibitor also significantly enhanced efficacy against p53-deficient tumors (Figures S7C–S7E) and increased T cell infiltration (Figures S7F and S7G) compared with anti-PD-1 alone, further demonstrating that MDM2 inhibition can effectively augment ICB efficacy, even in p53-deficient tumors.

## Discussion

In this study, to identify druggable post-translational regulators of necroptosis, we conducted a small-molecule screening centered on the ubiquitination machinery, utilizing a focused library directed at key components and regulators of the ubiquitination systems. With an early necroptosis readout and per-plate *Z* score normalization, approximately 40% of compounds exceeded the control threshold, consistent with the broad influence of ubiquitin dynamics on multiple steps of necroptotic signaling.^35^ Importantly, known necroptosis regulators appeared among the top hits, including mTOR (Torin-2)^21^ and SPOP (SPOP-IN-6IC),^22^ providing internal validation and aligning with prior reports. Within this framework, we uncovered MDM2 as a previously unrecognized negative regulator of TNF-α-driven necroptosis. Subsequent genetic ablation or pharmacologic inhibition of MDM2 markedly augmented TNF-α-induced necroptosis, and this enhancement was fully reversed by RIPK1 kinase inhibition or genetic inactivation, thereby establishing RIPK1 dependence and confirming the role of MDM2 regulating necroptosis.

While MDM2 is best known for mediating K48-linked ubiquitination and degradation of p53, thus suppressing apoptosis,^16^^,^^36^^,^^37^^,^^38^ recent evidence indicates that its regulatory repertoire also includes other forms of cell death, such as ferroptosis,^39^ suggesting that MDM2 is not limited to modulating p53 and apoptosis alone. Similarly, our data show that MDM2 regulates necroptosis independent of p53. Mechanistically, MDM2 interacts with RIPK3, with the N-terminal region of MDM2 binding the N-terminal kinase domain of RIPK3, then promotes the proteasomal degradation of RIPK3. Taken together, these findings support a model in which MDM2 suppresses necroptosis primarily by limiting RIPK3 abundance, rather than broadly altering NF-κB signaling. Given that RIPK3 is essential for necroptosis and that several E3 ligases, including PELI1, CHIP, and TRIM25,^12^^,^^40^^,^^41^ have been shown to promote its K48-linked ubiquitination, these observations support the view that ubiquitination of RIPK3 is a critical regulatory node for necroptotic signaling. In this context, we show that MDM2 is another key regulator of RIPK3, thereby acting as a molecular switch that tunes necroptosis and influences cell-fate decisions, and further expanding the set of E3 ligases linked to TNF-α-induced necroptosis.

MDM2 is widely recognized as a major oncogene. In the MSK-IMPACT clinical sequencing cohort, MDM2 amplification ranks as the seventh most frequent copy number alteration across cancers. To disrupt MDM2-p53 interaction, numerous agents have been developed and are undergoing clinical evaluation.^42^ However, most conventional anticancer drugs primarily target apoptosis, which is largely immunologically silent. By contrast, necroptosis not only leads to tumor cell death but also releases immunostimulatory signals within the TME, thereby coupling tumor-cell death to antitumor immunity.^5^ In this study, we demonstrated that MDM2 functions as a conserved suppressor of necroptosis across species, as genetic depletion or pharmacological inhibition of MDM2 enhanced necroptosis in both murine (E.G7) and human (SW480) tumor cells. These findings indicate that MDM2-mediated regulation of necroptosis is not restricted to a single tumor model but represents a broadly conserved mechanism in cancer cells. Consistent with this notion, single-cell transcriptomic profiling of tumor-infiltrating immune cells revealed that MDM2 deficiency is associated with a reduction in immunosuppressive myeloid subsets and enrichment of inflammatory gene programs, accompanied by an increased abundance and activation of cDC1 as well as enhanced CD8^+^ T cell infiltration. Importantly, pharmacological inhibition of necroptosis significantly diminished CD8^+^ T cell infiltration, indicating that MDM2-deficiency-driven necroptosis acts not merely as a tumor cell-intrinsic death pathway but as an immunogenic trigger that activates the tumor immune microenvironment and amplifies CD8^+^ T cell-mediated tumor killing.

In recent years, ICB, which aims to reverse CD8^+^ T cell exhaustion, has shown remarkable efficacy across multiple cancer types. However, many tumors, such as triple-negative breast cancer, glioblastoma, and pancreatic ductal adenocarcinoma, remain unresponsive to ICB, mostly sharing a strongly immunosuppressive, “cold” microenvironment.^43^^,^^44^^,^^45^ Prior studies have identified MDM2/MDM4 amplification as a predictor of poor response to anti-PD-1 therapy in MSKCC cohorts, and we further validated this association in additional datasets (Figures 6A and 6B). By demonstrating that MDM2 restrains tumor cell necroptosis, we not only provide a mechanistic explanation for MDM2-driven resistance toward ICB, but more importantly, propose a therapeutic strategy combining MDM2 inhibition with ICB, whereby MDM2 blockade increases tumor immunogenicity, inflames the TME, and improves responsiveness to anti-PD-1 therapy.

Notably, although MDM2 inhibitors were originally considered mainly for tumors with WT TP53, our findings reveal a second, p53-independent arm that suppresses necroptosis, thus broadening their potential use. In this framework, combining MDM2 inhibition with ICB to convert “cold” tumors into a T cell-inflamed microenvironment could benefit patients, irrespective of TP53 status, especially those with MDM2 amplification, and substantially enhance antitumor efficacy.

### Limitations of the study

Several limitations of this study should be acknowledged. First, our data demonstrated that MDM2 regulates RIPK3 protein stability under resting conditions; however, whether this regulatory axis is dynamically modulated by necroptotic signaling remains unclear. It is possible that necroptotic stimuli may influence MDM2-mediated control of RIPK3 turnover, either reinforcing or attenuating this regulation under specific contexts. Further investigation will be required to determine how necroptotic signaling feeds back onto MDM2-RIPK3 interactions in different cellular states.

Second, the *in vivo* experiments were performed using the E.G7-OVA tumor model, which represents an immunogenic transplant system. While suitable for mechanistic interrogation, this model may not fully recapitulate endogenous tumor development or the complexity of spontaneous tumor-immune interactions. Validation in additional tumor models would strengthen the generalizability of the findings.

Finally, whether the MDM2-RIPK3 regulatory axis operates similarly across diverse tumor types and microenvironmental contexts remains to be determined. Future studies exploring this pathway in additional tumor settings will help clarify its broader biological and therapeutic relevance.

## Resource availability

### Lead contact

Requests for further information and resources should be directed to and will be fulfilled by the lead contact, Xin Lin (linxin307@tsinghua.edu.cn).

### Materials availability

This study did not generate unique reagents.

### Data and code availability

• Data: Data reported in this paper will be shared by the lead contact upon request. All data associated with this study are provided in the manuscript or supplemental information. The mass spectrometry proteomics data have been deposited to the ProteomeXchange Consortium (https://proteomecentral.proteomexchange.org) via the iProX partner repository^46^^,^^47^ with the dataset identifier PXD075242. Raw and processed scRNA-seq data have been deposited in the Sequence Read Archive (SRA) and are publicly available with the dataset identifier PRJNA1426884. Both identifiers are listed in the key resources table (deposited data).
• Code: This paper does not report original code.
• Any additional information required to reanalyze the data reported in this paper is available from the lead contact upon request.

## Acknowledgments

We thank the Tsinghua University Laboratory Animal Research Center for animal husbandry and the Technology Center for Protein Sciences of Tsinghua University for assistance with mass spectrometry analyses. We are grateful to support of grants from 10.13039/501100012166National Key Research and Development Program of China (2020YFA0509101 and 2019YFA0508502 to X.L.), 10.13039/501100001809National Natural Science Foundation of China (31930039, 82293664, and 3182100 to X.L. and 81801576 to W.L.), Special Funding from the 10.13039/501100002858China Postdoctoral Science Foundation (2021T140373 to W.L.), and annual fund of Tsinghua-Peking 10.13039/501100011620Center for Life Sciences.

## Author contributions

Conceptualization, H.T., X.L., Y.W., and H.Y.; methodology, Y.W., W.X., and H.Y.; investigation, Y.W. and H.Y.; visualization, Y.W., H.Y., and Z. Zhang; bioinformatics analysis, H.Y., Z. Zhang, and Z. Zeng; funding acquisition, X.L. and W.L.; supervision, X.L. and H.T.; writing – original draft, Y.W.; writing – review & editing, H.Y. and X.L.

## Declaration of interests

The authors declare no competing interests.

## STAR★Methods

### Key resources table

**Table:** 

REAGENT or RESOURCE	SOURCE	IDENTIFIER
**Antibodies**		

RIP (D94C12) Rabbit Monoclonal Antibody	Cell Signaling Technology	Cat# 3493; RRID: AB_2305314
RIP3 (D4G2A) Rabbit Monoclonal Antibody (targeting mouse)	Cell Signaling Technology	Cat# 95702; RRID: AB_2721823
RIP3 (E7A7F) Rabbit Monoclonal Antibody (targeting human)	Cell Signaling Technology	Cat# 10188; RRID: AB_2904619
MLKL (D6W1K) Rabbit Monoclonal Antibody	Cell Signaling Technology	Cat# 37705; RRID: AB_2799118
Cleaved Caspase-3 (Asp175) (5A1E) Rabbit Monoclonal Antibody	Cell Signaling Technology	Cat# 9664; RRID: AB_2070042
Caspase-3 (8G10) Rabbit Monoclonal Antibody	Cell Signaling Technology	Cat# 9665L RRID: AB_2069872
p53 (1C12) Mouse Monoclonal Antibody	Cell Signaling Technology	Cat# 2524; RRID: AB_331743
DYKDDDDK Tag Antibody (Binds to same epitope as Sigma-Aldrich Anti-FLAG M2 antibody)	Cell Signaling Technology	Cat# 2368S; RRID: AB_2217020
Anti-MDM2 antibody [EPR22256-98]	Abcam	Cat# ab259265; RRID: AB_2920616
Anti-MLKL (phospho S345) antibody [EPR9515(2)] (targeting mouse)	Abcam	Cat# ab196436; RRID: AB_2687465
Anti-MLKL (phospho S358) antibody [EPR9514] (targeting human)	Abcam	Cat# ab187091; RRID: AB_2619685
Anti-Ubiquitin (linkage-specific K48) antibody [EP8589]	Abcam	Cat# ab140601; RRID: AB_2783797
RIP3 Antibody (B-2) (for immunoprecipitation)	Santa Cruz Biotechnology	Cat# sc-374639; RRID: AB_10992232
PCNA Antibody (PC10)	Santa Cruz Biotechnology	Cat# sc-56; RRID: AB_628110
NF-κB p65 Antibody (A)	Santa Cruz Biotechnology	Cat# sc-109; RRID: AB_632039
Anti-β-tubulin Mouse Monoclonal Antibody	Easy Bio.	Cat# BE0025-100; RRID: AB_3718711
Anti-GAPDH Mouse Monoclonal Antibody	Easy Bio.	Cat# BE0023; RRID: AB_3665256
Bax Rabbit mAb	Selleck	Cat# F0037; RRID: AB_3698184
PUMA Rabbit mAb	Selleck	Cat# F0558
PARP1 Antibody [N4A5]	Selleck	Cat# F0148; RRID: AB_3722983
PE-Cy7 conjugated anti-CD3	Biolegend	Cat# 100220; RRID: AB_1732057
Alexa Fluor 700 conjugated anti-CD4	Biolegend	Cat# 116022; RRID: AB_2715958
PE/Cyanine7 conjugated anti-CD11c	Biolegend	Cat# 117318; RRID: AB_493568
FITC conjugated anti-Ly6G	BD	Cat# 551460; RRID: AB_394207
Alexa Fluor 700 conjugated anti-Ly6C	BD	Cat# 561237; RRID: AB_10612017
PE conjugated anti-CD86	BD	Cat# 553692; RRID: AB_394994
APC-eFluor 780 conjugated anti-CD8	eBioscience	Cat# 47-0081-82; RRID: AB_1272185
PerCP-Cyanine5.5 conjugated anti-CD45.2	eBioscience	Cat# 45-0454-82; RRID: AB_953590
APC-eFluor 780 conjugated CD45.2	eBioscience	Cat# 47-0454-82; RRID: AB_1272175
PerCP-Cyanine5.5 conjugated anti-CD11b	eBioscience	Cat# 45-0112-82; RRID: AB_953558
PE-conjugated anti-F4/80	eBioscience	Cat# 12-4801-82; RRID: AB_465923
APC-conjugated anti-MHC Class II	eBioscience	Cat# 17-5320-82; RRID: AB_2573212
FITC conjugated anti-CD80	eBioscience	Cat# 11-0801-82; RRID: AB_465133
V500-conjugated Cell viability Dye	eBioscience	65-0866-18
Rat IgG2b isotype control-InVivo	Selleck	Cat# A2116; RRID: AB_3662740
Anti-mouse CD8α-InVivo	Selleck	Cat# A2102; RRID: AB_3099521
InVivoMab anti-mouse PD-1 (CD279)	BioXcell	Cat# BE0146; RRID: AB_10949053

**Bacterial and virus strains**		

DH5a chemically Competent Cell	Homemade	N/A
Lentivirus	This paper	N/A

**Chemicals, peptides, and recombinant proteins**		

Recombinant Mouse TNF-alpha (aa 80–235) Protein	R&D	Cat# 410-MT
TNF-α (soluble) (human), (recombinant)	Enzo life science	Cat# ALX-522-008
BV-6	Selleck	Cat# S7597
GSK’872	Selleck	Cat# S8465
MG132	Selleck	Cat# S2619
Chloroquine	Selleck	Cat# S6999
Nutlin-3a	Selleck	Cat# S1061
Necrostatin-1	Beyotime	Cat# N9037
zVAD.fmk	Beyotime	Cat# C1202
DNase I	Sigma-Aldrich	Cat# DN25
Collagenase Type II	Gibco	Cat# 17101015

**Critical commercial assays**		

CytoTox 96 Non-Radioactive Cytotoxicity Assay	Promega	Cat #G1780

**Deposited data**		

mass spectrometry proteomics data	This paper	iProX^46^^,^^47^: PXD075242
scRNA-seq data	This paper	Sequence Read Archive (SRA); SRA: PRJNA1426884

**Experimental models: Cell lines**		

Lenti-X 293T	Takara	Cat# 632180
L929	ATCC	Cat# CCL-1
HEK293T	ATCC	Cat# CRL-11268
Raw 264.7	ATCC	Cat# TIB-71
iBMDM	Homemade	N/A
SW480	procell	Cat# CL-0223
E.G7-OVA	eallbio	Cat# 06.0272
B16	procell	Cat# CL-0029

**Experimental models: Organisms/strains**		

C57BL/6 WT mice	Tsinghua University	N/A
*Batf3*^−/−^ mice	The Jackson Laboratory	Cat# 013755

**Oligonucleotides**		

Oligonucleotide list, see Generation of CRISPR-Cas9-edited cell lines below	This paper	N/A

**Recombinant DNA**		

pEGFP-C1-MDM2	This paper	N/A
pEGFP-C1-N′-MDM2	This paper	N/A
pEGFP-C1-C′-MDM2	This paper	N/A
pCMV-FLAG-RIPK3	This paper	N/A
pCMV-FLAG- N′-RIPK3	This paper	N/A
pCMV-FLAG- C′-RIPK3	This paper	N/A
pCMV-FLAG-RIPK1	This paper	N/A
Lenti-CRISPR-V2-NT	This paper	N/A
Lenti-CRISPR-V2-mMDM2 sg1	This paper	N/A
Lenti-CRISPR-V2-mMDM2 sg2	This paper	N/A
Lenti-CRISPR-V2-mp53-sg1	This paper	N/A
Lenti-CRISPR-V2- mp53-sg2	This paper	N/A
Lenti-CRISPR-V2- hMDM2-sg1	This paper	N/A
Lenti-Cas9-BSD	This paper	N/A
Lenti-mMDM2 sg1-Efs-EGFP	This paper	N/A
Lenti-mMDM2 sg2-Efs-EGFP	This paper	N/A

**Software and algorithms**		

Prism 10.0	GraphPad	N/A
FlowJo	FlowJo LLC	N/A
ImageJ	National Institutes of Health	N/A
Xcalibur 3.0 software	Thermo Fisher Scientific	N/A

### Experimental model and study participant details

#### Mice

All mice were housed in the specific pathogen-free (SPF) animal facilities at Tsinghua University. Male and female C57BL/6 WT mice were purchased from Tsinghua University, female *Batf3*^−/−^(JAX stock no.013755) mice were kindly provided by Yangxin Fu’s lab (Tsinghua University). All mice used in the study aged 6 to 8 weeks at the time of experimentation. All mouse experiments were performed in compliance with institutional guidelines and according to the protocol approved by the Institutional Animal Care and Use Committee (IACUC) of Tsinghua University (ethical approval number: 15-LX1).

#### Cell culture

All cells were cultured at 37°C and 5% CO_2_. HEK293T (purchased from ATCC), L929, SW480, B16, were cultured in DMEM medium (GIBCO) and E.G7-OVA cells were cultured in RPMI 1640 medium (GIBCO) supplemented with 10% FBS, non-essential amino acids, sodium pyruvate, penicillin, streptomycin. Cell lines were purchased, tested routinely for negative contamination of mycoplasma, as certified by the manufacturers, and were not further authenticated before use.

#### Generation of CRISPR-Cas9-edited cell lines

Different MDM2 and p53 knockdown or knock out cell lines were infected with sgRNA expressing lentivirus. sgRNA sequences were as follows:

sgControl:

oligo1: ggacgctaaaccaacggtgc; oligo2: gcaccgttggtttagcgtcc;

sg*Mdm2*-1 (mouse):

oligo1: ttgaagttgttaaagtccgt; oligo2: acggactttaacaacttcaa;

sg*Mdm2*-2 (mouse):

oligo1: gccagtatattatgactaag; oligo2: cttagtcataatatactggc;

sg*p53*-1 (mouse):

oligo1: aaaatgtctcctggctcaga; oligo2: tctgagccaggagacatttt;

sg *p53*-2 (mouse):

oligo1: agtgaagccctccgagtgtc; oligo2: gacactcggagggcttcact;

sg*MDM2* (human):

oligo1: agggtctcttgttccgaagc; oligo2: gcttcggaacaagagaccct.

### Method details

#### Cell infection and transfection

A lentiviral supernatant was collected 48 h after co-transfection of expression plasmids (Lenti-RFP-Puro, Lenti-MDM2-sg1- Puro, Lenti-MDM2-sg2-Puro, Lenti-p53-sg1- Puro, Lenti-p53-sg2-Puro, Lenti-sgControl-BSD, Lenti-p53-sg1- BSD, Lenti-p53-sg2-BSD, Lenti-Cas9-BSD, Lenti-MDM2 sg1-Efs-EGFP, Lenti-MDM2 sg2-Efs-EGFP) with packaging plasmids (psPAX2 and pMD2.G) into HEK293T cells, and the lentiviral supernatants were collected after 48 h. Target cells incubated with the viral supernatants in the presence of polybrene for 8–12 h, and then the virus supernatant was replaced with fresh medium. Successfully infected cells were selected by using puromycin or blasticidin after 48 h. The infection efficiency was detected by doing western blotting analysis.

Transient transfections of 293T cells were performed using PEI according to the manufacturer’s instructions. In brief, 293T cells were seeded in 6-well plates. When cells were 95% confluent, plasmids were transfected using PEI reagent at a ratio of 1:3, and each well was transfected with a total of 2 μg DNA per well for 24 h.

#### Cytotoxicity assays

The dead tumor cells were determined by lactate dehydrogenase (LDH)- based assays (CytoTox 96 Non-Radioactive Cytotoxicity Assay, Promega #G1780). L929, SW480 cells were seeded the day before at 3×10^4^ per well while E.G7-OVA at 1×10^5^ per well in duplicates in a 96-well plate. The next day, after indicated stimulation, the released LDH in the supernatant was measured with a coupled enzymatic assay, resulting in the conversion of a tetrazolium salt into a red formazan product. The strength of red color is proportional to the number of lysed cells and could be determined by standard plate reader (800 TS, BioTek). Percent cytotoxicity = 100 × (experimental LDH Release (OD490))/maximum LDH Release (OD490).

#### Western-blotting, immunoprecipitation, and ubiquitination assays

Cells were lysed in lysis buffer (50 mM HEPES, pH 7.4, 150 mM NaCl, 1% NP-40, 1 mM EDTA) containing 1 mM sodium orthovanadate, 1 mM sodium fluoride, 1 mM phenylmethylsulfonyl fluoride (PMSF), and a protease inhibitor mixture (Roche). Cell lysates were then subjected to sodium dodecyl sulfate-polyacrylamide gel electrophoresis (SDS-PAGE) and transferred onto a polyvinylidene difluoride membrane (Millipore). The membrane was sequentially probed with primary antibodies and HRP-conjugated secondary antibodies. ECL substrates (Pierce) were used to visualize the specific bands on the membrane. Original blots are provided in the Source Data file.

For Co-immunoprecipitation, cell lysates of 293T cells were incubated with Anti-Flag Affinity Gel (B23102, Bimake-Selleck) for 3h at 4 °C. After extensive washes, beads were boiled in loading buffer and eluted products were separated by SDS-PAGE, which were transferred to PVDF membrane and analyzed with indicated antibodies.

For ubiquitination assay, cells were lysed in RIPA lysis buffer (50 mM Tris, pH 7.4, 150 mM NaCl, 1 mM EDTA, 20 mM N-ethylmaleimide, and 1% Triton X-100). Lysates were immediately boiled for 10 min in the presence of 1% (vol/vol) SDS and then were diluted with a lysis buffer until the concentration of SDS was decreased to 0.1%. Immunoprecipitates were analyzed by immunoblot with anti- K48 ubiquitin antibody.

#### Nuclear extraction

1 × 10^6^ L929 cells were lysed in nuclear lysis buffer (10 mM HEPES, pH 7.9, 10 mM KCl, 0.1 mM EDTA and 0.4% NP40) containing protease inhibitors for 30min on ice. The pellet (intact nuclei) was collected by centrifuging at 18,000 × g for 15 min at 4 °C and washed in nuclear lysis buffer. After washing for at least 5 times, the pellet was lysed in nuclear extraction buffer (20 mM HEPES, pH 7.9, 0.4M NaCl and 1 mM EDTA) containing protease inhibitors for 2h at 4 °C. The nuclear extracts were collected in the supernatants by centrifuging at 18,000 × g for 15 min at 4 °C.

#### BMDM isolation

Bone marrow was flushed from the tibia and femurs of 6-week-old mice and lysed by RBC buffer. The bone marrow-derived macrophage (BMDM) cells were cultured in DMEM supplemented with 20% M-CSF-containing conditional medium from L929 cells, penicillin-streptomycin and 15% FBS for 7 days. All cells were cultured at 37°C and 5% CO2.

#### Flow cytometry analysis

For cell surface marker staining, cultured-cell lines or single-cell suspensions isolated from tumor tissue were used for staining cell surface markers following standard protocols. Antibodies for flow cytometry were used with 200- to 500-fold dilution. For intracellular staining, cells were processed using the Cytofix/Cytoperm Kit per manufacturer’s instructions (BD Biosciences, #554715) and stained with indicated antibodies. Data acquisition was performed using FACSAria II cytometer (BD). Flow cytometric data were analyzed with the FlowJo software.

#### Tumor inoculation

For *in vivo* tumor model, male 6-8-week-old C57BL/6 J mice were subcutaneously injected with the indicated cell lines and cell numbers. Tumor cells suspensions were mixed with Matrigel (BD Biosciences, #356234) at 1:1 ratio by volume, and then subcutaneously injected into the flank. The solid tumor was measured every two days and the tumor volume was calculated as (length× widthˆ2)/2. Mice were euthanized if they exhibited symptoms of distress or when tumor size reached ethical endpoint of 2000 mm^3^.

#### Primary tumor tissue extraction

Primary tumor tissues were harvested and frozen with liquid nitrogen. Then the tumor tissue was ground and lysed with RIPA buffer (Beyotime, P0013B) plus protease inhibitors for 2h at 4 °C. Whole-cell lysate was collected by centrifuge at 18,000 × g for 15 min at 4 °C and the protein concentration was detected by Bradford Protein Concentration Test Kit (Beyotime, P0006).

### Quantification and statistical analysis

#### Mass spectrometry and data analysis

For mass spectrometry on whole proteomic detection, the gel bands of interest were excised from the gel, which was followed by in-gel digestion with sequencing grade modified trypsin at 37°C overnight. The peptides were extracted twice with 0.1% trifluoroacetic acid in 50% acetonitrile aqueous solution for 30 min and then dried in a speedvac. Peptides were redissolved in 25 μL 0.1% trifluoroacetic acid and 6 μL of extracted peptides were analyzed by Thermo orbitrap fusion. For LC-MS/MS analysis, the peptides were separated by a 60 min gradient elution at a flow rate 0.30 μL/min with an EASY-nLC 1000 system, which was directly interfaced with an Orbitrap Fusion Tribrid mass spectrometer (Thermo Fisher Scientific, Bremen, Germany). The analytical column was a home-made fused silica capillary column (75 μm ID, 150 mm length; Upchurch, Oak Harbor, WA) packed with C-18 resin (300 Å, 5 μm, Varian, Lexington, MA). Mobile phase consisted of 0.1% formic acid, and mobile phase B consisted of 100% acetonitrile and 0.1% formic acid. The Orbitrap Fusion mass spectrometer was operated in the data-dependent acquisition mode using Xcalibur3.0 software and there was a single full-scan mass spectrum in the orbitrap (350–1550 m/z, 120,000 resolution) followed by top-speed MS/MS scans in the Orbitrap.

#### Single-cell RNA sequencing (scRNA-seq)

Live CD45^+^ immune cells were sorted from both NT and MDM2-deficient E.G7 murine tumor models. For each group, equal number of CD45^+^ immune cells from two different mice were mixed together. Single-cell RNA-seq libraries were prepared using SeekOne DD Single Cell 3′ library preparation kit (SeekGene). The libraries were sequenced on Illumina NovaSeq 6000 with PE150 read length. Raw sequencing data were processed by SeekOne tools and further analyzed by Seurat (v4.0.0). The variable genes were calculated by FindVariableGenes. Then, all libraries were combined together using FindIntegrationAnchors and IntegrateData with defaut parameters, and using ScaleData to regress out the variability of the numbers of UMIs. Then the RunPCA and RunUMAP was used to reduce dimensions and FindAllMarkers was used to compare each cluster to all others to identify cluster-specific marker genes.

#### Statistical analysis

Statistical analyses were performed using GraphPad Prism 10 software. No statistical methods were used to predetermine sample size. Statistical comparisons between multiple groups were determined by one-way analysis of variance (ANOVA) or two-way ANOVA, and Tukey’s multiple comparison test was used. Survival curves were generated using the Kaplan-Meier method, and statistical significance between groups was assessed using the log rank test. *p* < 0.05 was considered to be statistically significant. The statistical test used for each Figure is described in the corresponding Figure legend.

## Supplemental information

Document S1. Figures S1–S7

Table S1. List of differential proteins in MDM2-deficient L929 cells Differential proteomic analysis comparing MDM2-deficient and control L929 cells. The table lists significantly altered proteins identified by mass spectrometry, together with log_2_ FC, average expression, *t* value, *p* value, adjusted *p* value, and direction of change. Positive indicates proteins upregulated in MDM2-deficient cells relative to control cells.
