# Supplementary material for: Inhibiting MDM2 enhances RIPK3-mediated necroptosis and synergizes with immune checkpoint blockade therapy
Source: iScience. 2026 Apr 11;29(5):115724. doi: 10.1016/j.isci.2026.115724 (PMC13141637; doi:10.1016/j.isci.2026.115724)
Supplement: Document S1. Figures S1–S7 [file mmc1.pdf]

iScience, Volume *29*

Supplemental information

Inhibiting MDM2 enhances RIPK3-mediated necroptosis and synergizes with immune checkpoint blockade therapy

Yingxin Wu, Hanyang Yu, Zongxu Zhang, Weihang Xiong, Zexian Zeng, Weili Liu, Hailin Tu, and Xin Lin

Supplemental Materials

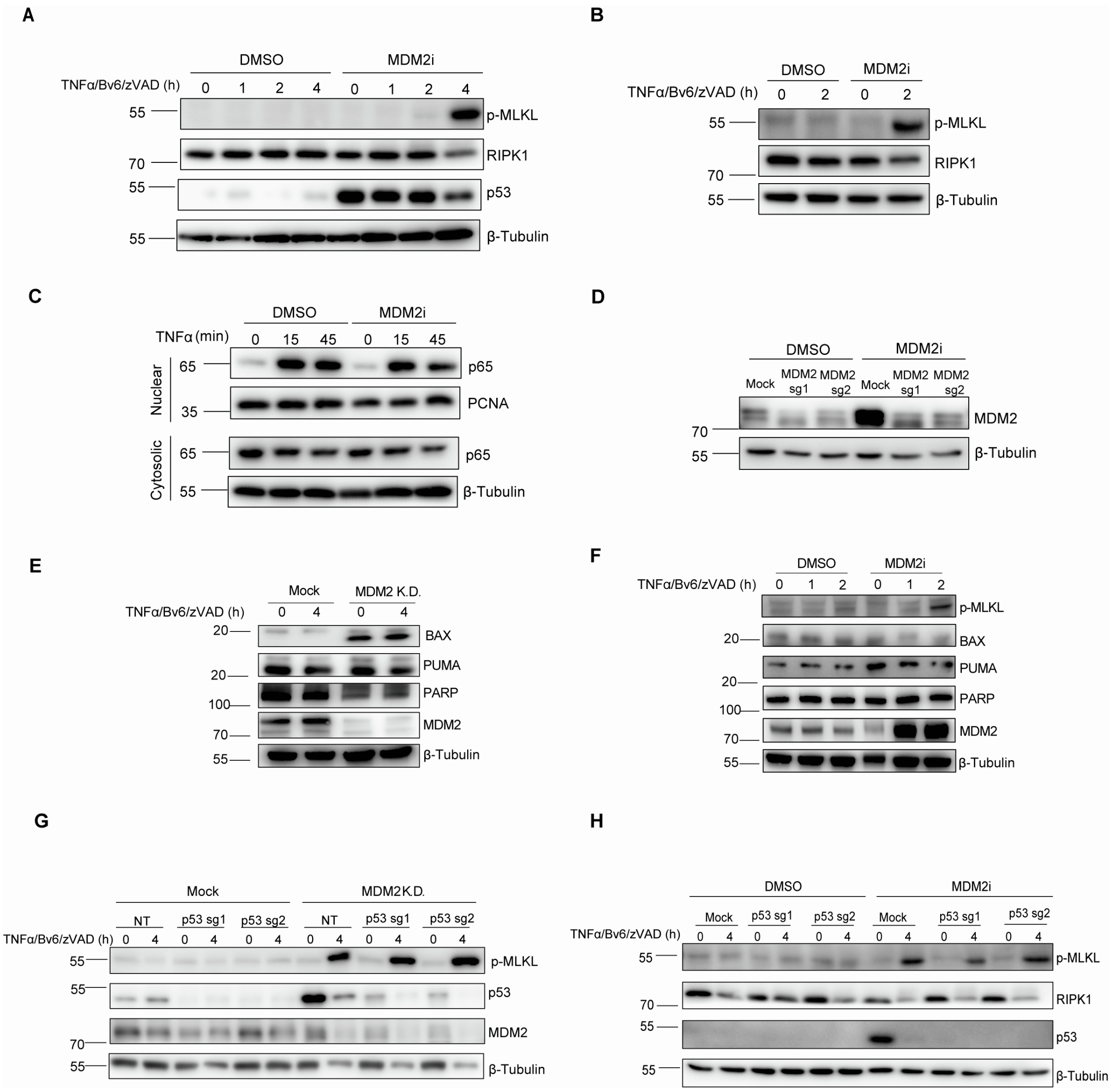

Figure S1. MDM2 inhibition and deficiency universally promote RIPK1-dependent necroptosis in a p53-independent way, related to Figure 2.

(A and B) (**A**) Raw264.7 and (**B**) iBMDM cells were treated with DMSO or MDM2 inhibitor Nutlin-3a followed by T/B/Z treatment for indicated time points. The cell lysates were analyzed by western blotting using indicated antibodies.

(C) L929 treated with DMSO or Nutlin-3a were stimulated with TNFα (20 ng/mL) at indicated times, then the nucleus was extracted and analyzed by western blotting with antibodies against p65 and PCNA.

(D) Knockout efficiency of MDM2 in L929 was evaluated by western blot with antibodies against MDM2. The cells were treated with Nutlin-3a to enhance the expression of MDM2.

(E) Mock and MDM2 K.D. L929 cells were treated with T/B/Z at indicated time points. The cell lysates were analyzed by western blotting using indicated antibodies.

(F) L929 cells were treated with DMSO or Nutlin-3a followed by T/B/Z treatment for indicated time points. The cell lysates were analyzed by western blotting using indicated antibodies.

(G) p53 K.D. was engineered by CRISPR-Cas9 in Mock and MDM2 K.D. L929 cells then stimulated with T/B/Z for 4 h. The cell lysates were analyzed by western blotting using indicated antibodies.

(H) p53 K.O. was engineered by CRISPR-Cas9 in L929 cells then stimulated with DMSO or Nutlin-3a followed by T/B/Z treatment for indicated time points. The cell lysates were analyzed by western blotting using indicated antibodies. TNFα, 10 ng/mL; Bv6, 2.5 μM; zVAD, 20 μM.

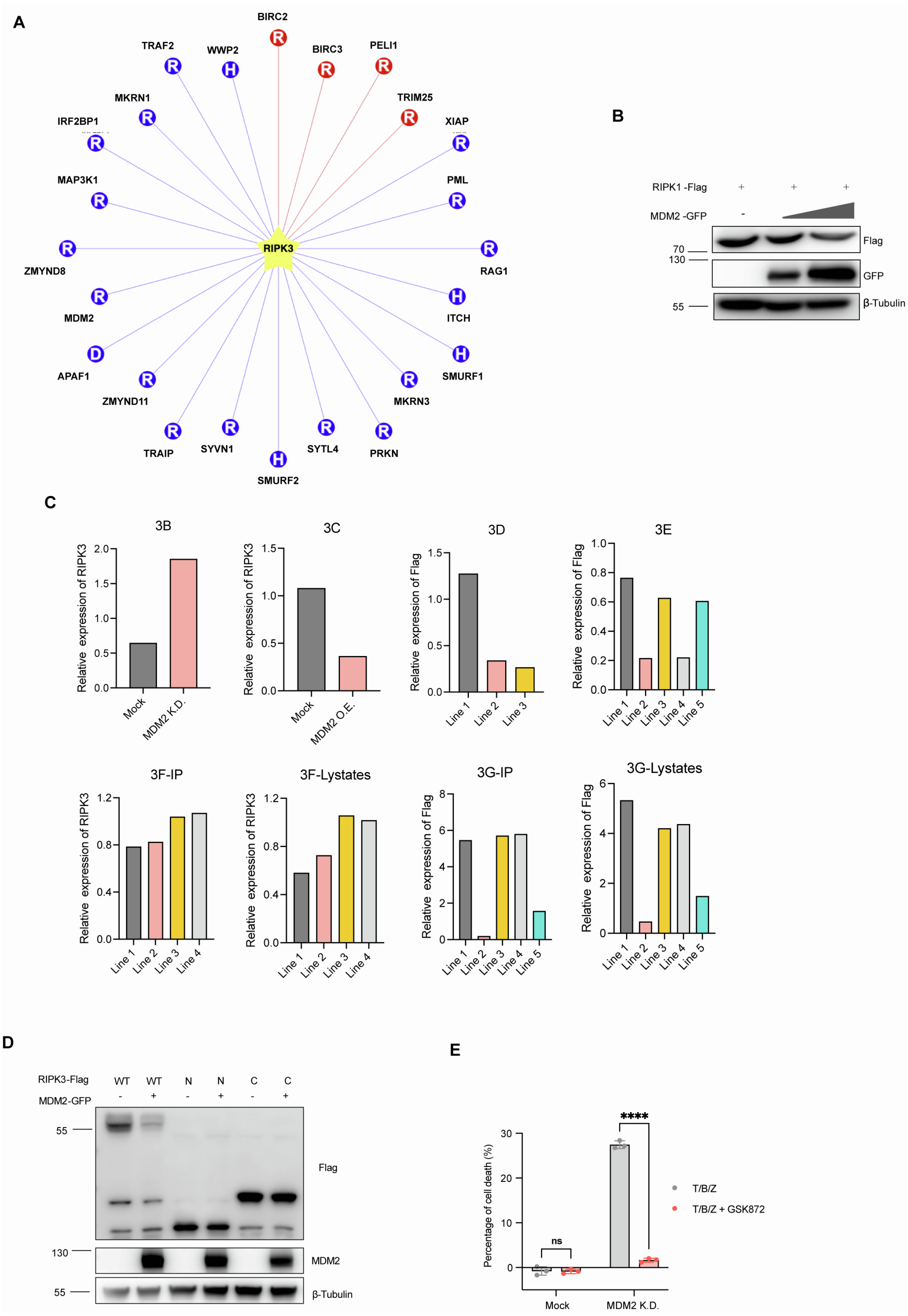

Figure S2. MDM2 promotes the degradation of RIPK3 thus inhibiting necroptosis, related to Figure 3.

(A) Potential E3 ligases targeting RIPK3 predicted by UbiBrowser including 4 known and 20 top predicted E3 ligases. Red means known E3 ligases while blue means

predicted E3 ligases. The predicted proteins all have ubiquitination functional domain including RING (R), HECT (H) or DWD (D).

(B) RIPK1-Flag plasmid was co-transfected with increased concentrations of MDM2-GFP in 293T cells for 24 h, then the cell lysates were analyzed by western blotting using indicated antibodies.

(C) Quantification of western blotting analyses shown in Fig. 3B-G. Relative protein expression levels were quantified by densitometry using ImageJ and normalized to β-Tubulin.

(D) 293T cells were transfected with the indicated Flag-tagged RIPK3 and MDM2-GFP. Western blotting was performed using the indicated antibodies.

(E) Mock and MDM2 K.D. L929 cells were treated with T/B/Z for 5 h in the presence or absence of RIPK3 inhibitor GSK'872. The cell death was determined by LDH release in the supernatant. TNFα, 10 ng/mL; Bv6, 2.5 μM; zVAD, 20 μM; GSK872, 10 μM; Data are represented as mean ± SEM. P values determined by two-way ANOVA with Tukey's correction for multiple comparisons. ****P < 0.0001; ns, not significant.

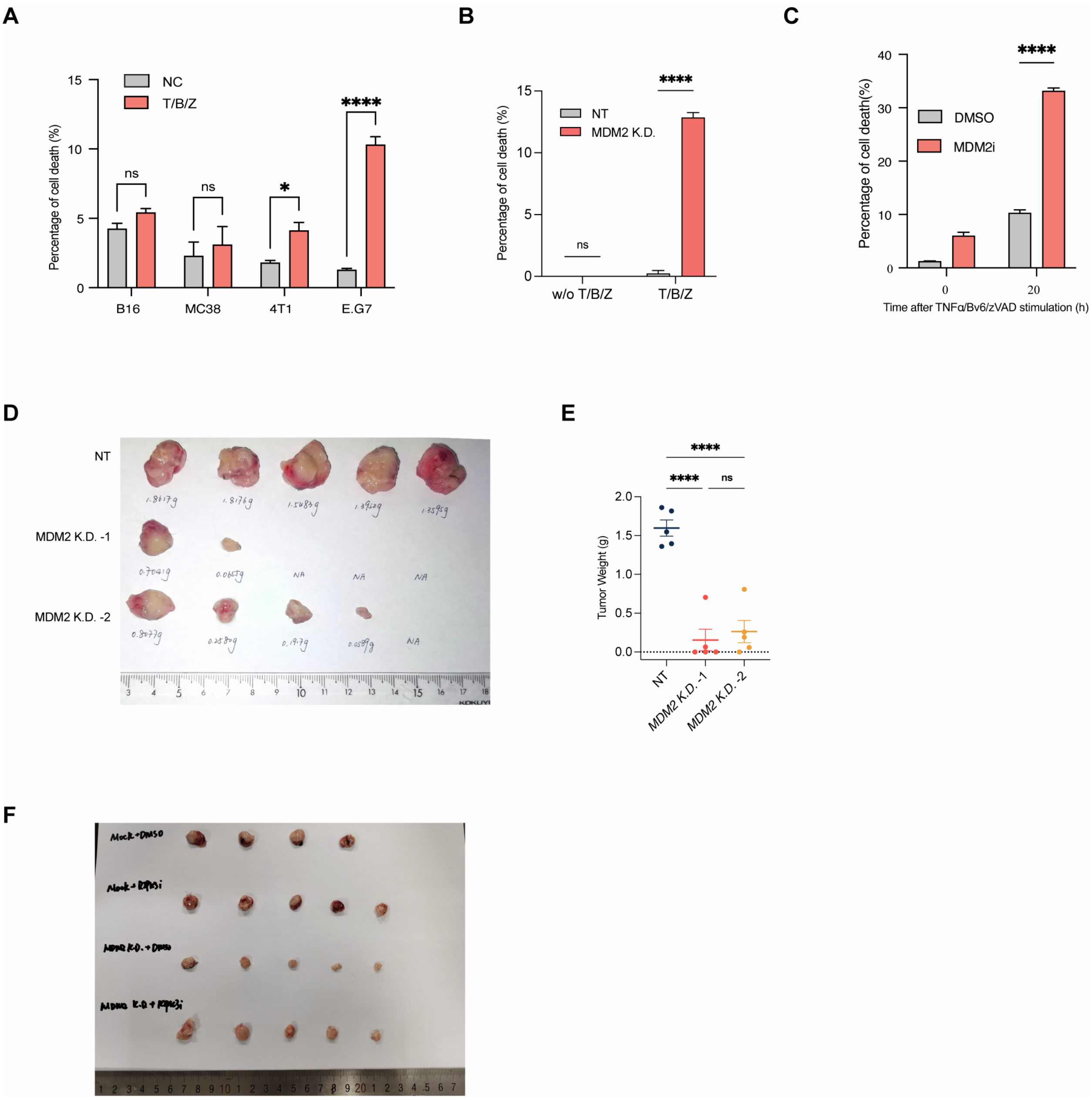

Figure S3. MDM2 deficiency induces necroptosis in vivo and in vitro in the E.G7 tumor model, related to Figure 4.

(A) B16, MC48, 4T1 and E.G7 cells were treated with T/B/Z for 36 h. Cell death was determined by LDH release in the supernatant.

(B) NT and MDM2 K.D. E.G7-OVA cells were treated with T/B/Z for 20 h. Cell death was determined by LDH release in the supernatant.

(C) E.G7 cells were treated with T/B/Z in the presence or absence of MDM2 inhibitor for 20h. Live cells were detected by flow cytometry after staining with viability dyes. **(D and E)** NT and MDM2 K.D. E.G7 tumors harvested from C57BL/6 mice on day 19, then (**D**) the tumors were photographed and (**E**) the weights of tumors were measured. **(F)** GSK'872 (10 μg/g) was injected intraperitoneally every day after day 5, and tumors were harvested on day 11 then photographed. TNFα, 160 ng/mL; Bv6, 2.5 μM; z-VAD, 20 μM. Data are represented as mean ± SEM. P values were determined by ordinary two-way ANOVA with Tukey's correction for multiple comparisons. $^{*}P < 0.05$, $^{****}P < 0.0001$; ns, not significant.

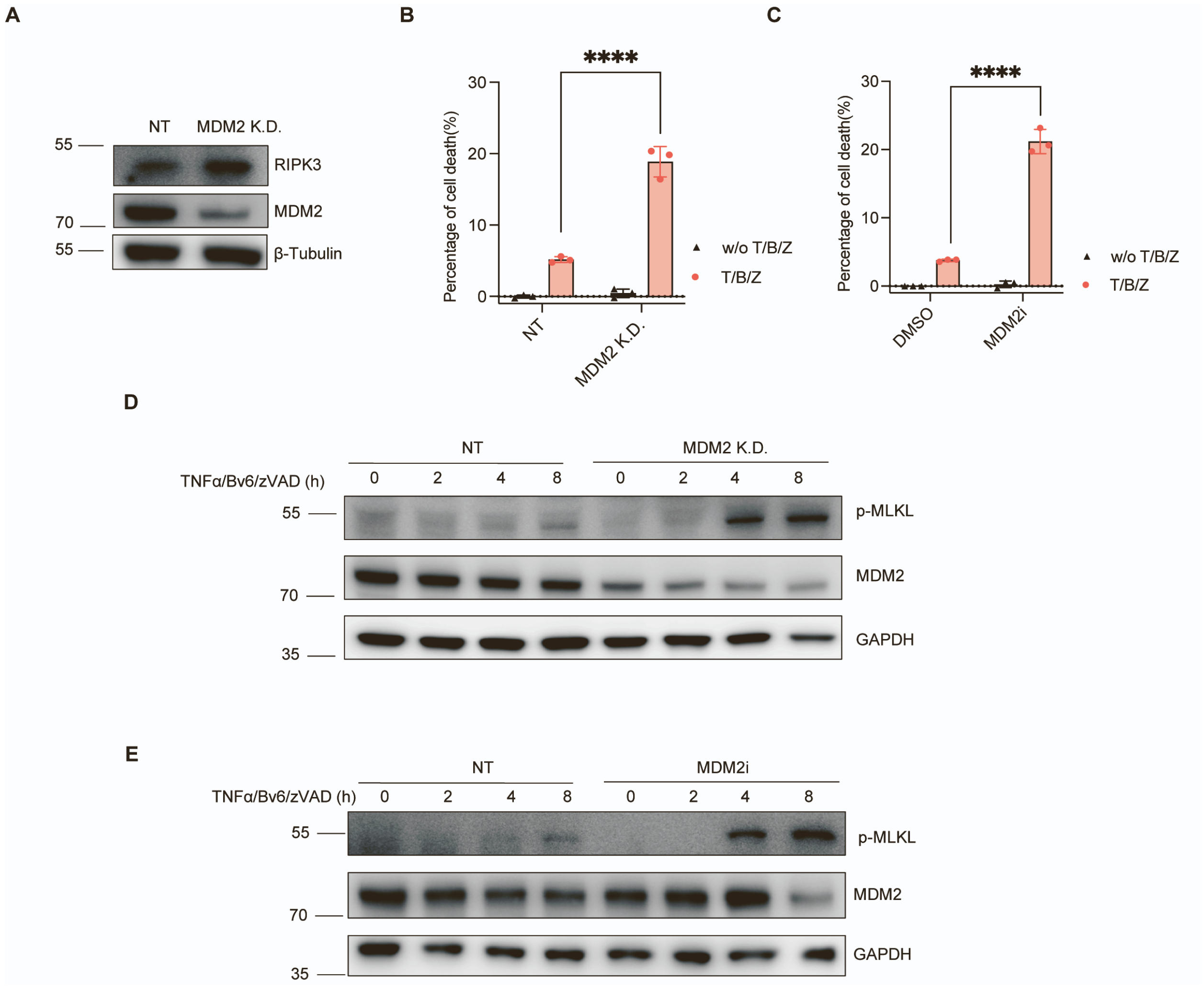

Figure S4. MDM2 promotes the degradation of RIPK3 thus inhibiting necroptosis in SW480, related to Figure 4.

(A) Levels of RIPK3 in NT and MDM2 K.D. SW480 cells were analyzed by western blotting.

(B) NT and MDM2 K.D. SW480 cells were treated with T/B/Z for 36 h. Cell death was determined by LDH release in the supernatant.

(C) SW480 cells were treated with DMSO or MDM2 inhibitor Nutlin-3a followed by T/B/Z treatment for 33 h. The cell death was determined by LDH release in the supernatant.

(**D**) NT and MDM2 K.D. SW480 cells were treated with T/B/Z at indicated time points. The cell lysates were analyzed by western blotting using indicated antibodies.

(**E**) SW480 cells were treated with DMSO or Nutlin-3a followed by T/B/Z treatment for indicated time points. The cell lysates were analyzed by western blotting using indicated antibodies. TNFα, 10 ng/mL; Bv6, 2.5 μM; zVAD, 20 μM; Data are represented as mean ± SEM. P values were determined by two-way ANOVA with Tukey's correction for multiple comparisons. ****$P < 0.0001$.

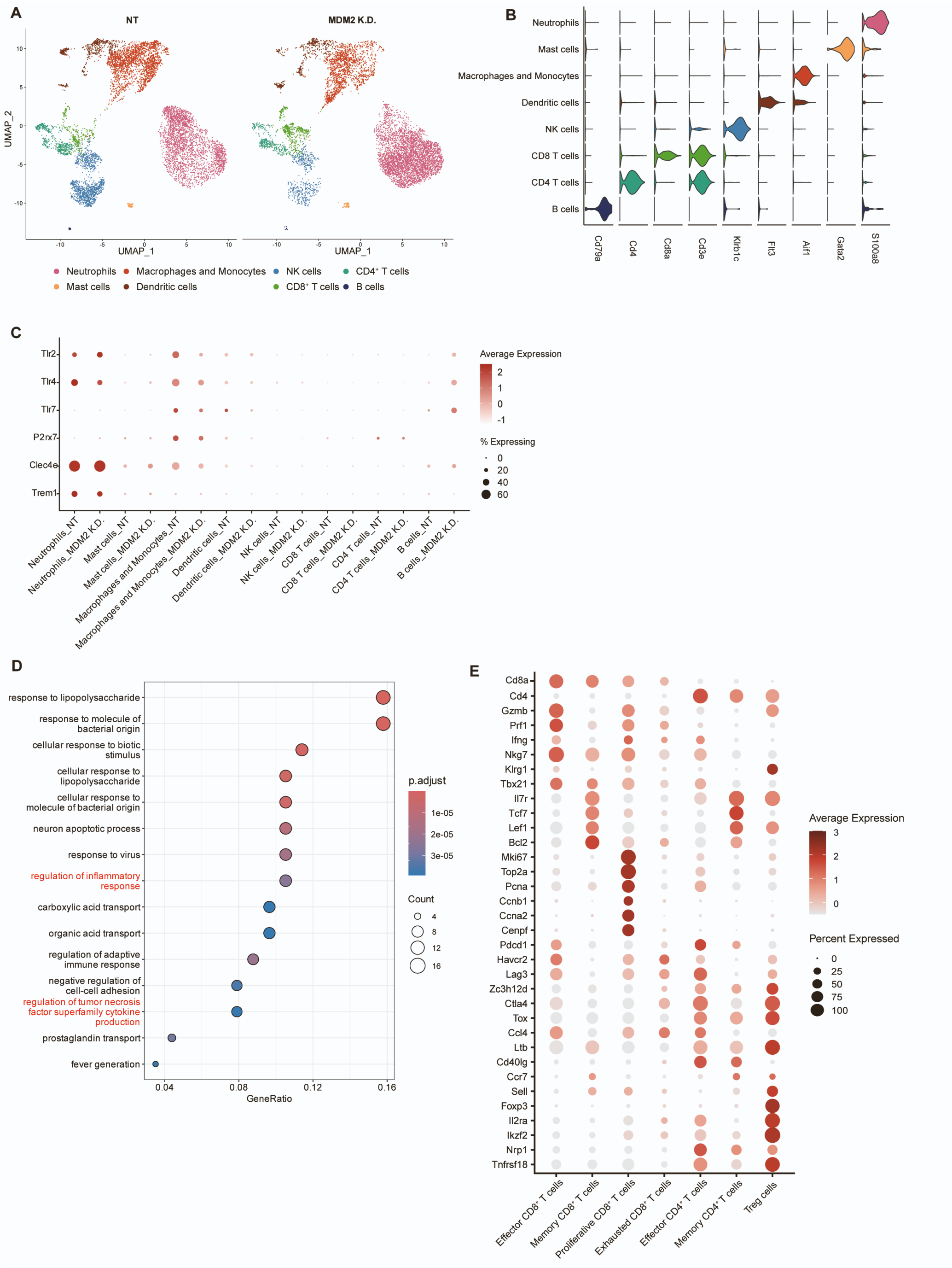

Figure S5. Single-cell transcriptomic profiling of immune cell composition and functional signatures in NT and MDM2 K.D. E.G7 tumors, related to Figure 4 and Figure 5.

(A) UMAP plot showing the overall immune cell landscape in NT and MDM2 K.D. tumors.

(B) Violin plots displaying the expression of representative marker genes used to define each immune cell population.

(C) Dot plot illustrating the expression levels and percentage of cells expressing key DAMP-related receptors across all immune cell types in NT and MDM2 K.D. tumors. Dot size represents the percentage of expressing cells, and color intensity indicates the average expression level.

(D) GO enrichment analysis of differentially expressed genes in myeloid cells from MDM2 K.D. tumors, highlighting pathways associated with inflammatory responses and immune activation.

(E) Dot plot showing the expression patterns of representative marker genes used to identify T cell subclusters in scRNA-seq data, including effector $CD8^+$ T cells, memory $CD8^+$ T cells, proliferative $CD8^+$ T cells, exhausted $CD8^+$ T cells, effector $CD4^+$ T cells, memory $CD4^+$ T cells, and regulatory T (Treg) cells. Dot size represents the percentage of cells expressing each gene within the cluster, and color intensity indicates the average expression level.

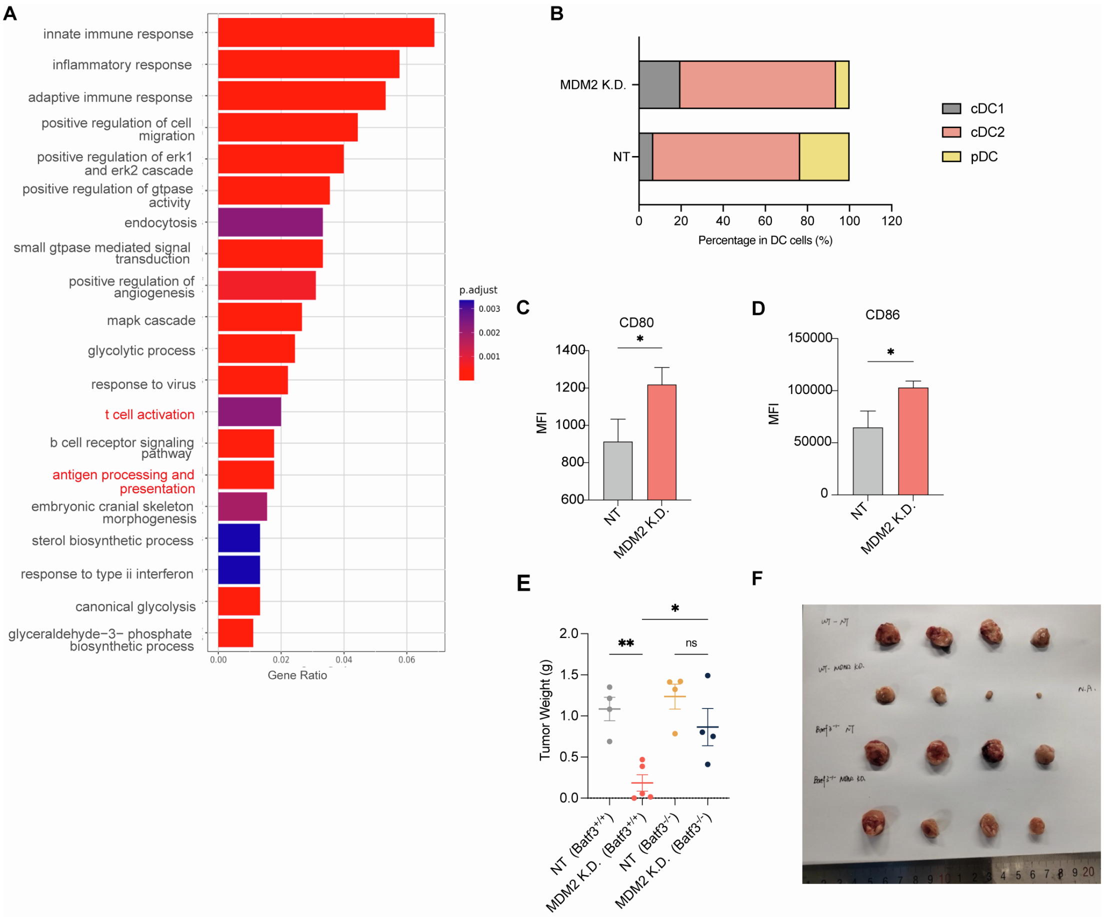

Figure S6. DC activation and functional validation in MDM2-deficient tumors, related to Figure 5.

(A) Gene Ontology biological process (GOBP) enrichment analysis of DCs from MDM2 K.D. tumors, highlighting pathways involved in adaptive immune activation and antigen presentation.

(B) Proportions of cDC1, cDC2, and pDC subsets among total DCs in NT and MDM2 K.D. E.G7 tumors based on single-cell RNA-seq analysis.

(C and D) Flow cytometry analysis of activation markers (**C**) CD80 and (**D**) CD86 on DCs in tumor-draining lymph nodes from NT and MDM2 K.D. mice. Data are shown as mean fluorescence intensity (MFI).

(E and F) Functional assessment of DC involvement in $CD8^+$ T cell infiltration using *Batf3*$^{-/-}$ mice, in which cDC1s are selectively absent. (**E**) Tumor weights and (**F**) representative tumor images are shown. Data are represented as mean ± SEM. P values in figure (**E**) were determined by ordinary two-way ANOVA with Tukey's correction for multiple comparisons, and (**C**), (**D**) were determined by ordinary one-way ANOVA. *P < 0.05, **P < 0.01, ***P < 0.001; ns, not significant.

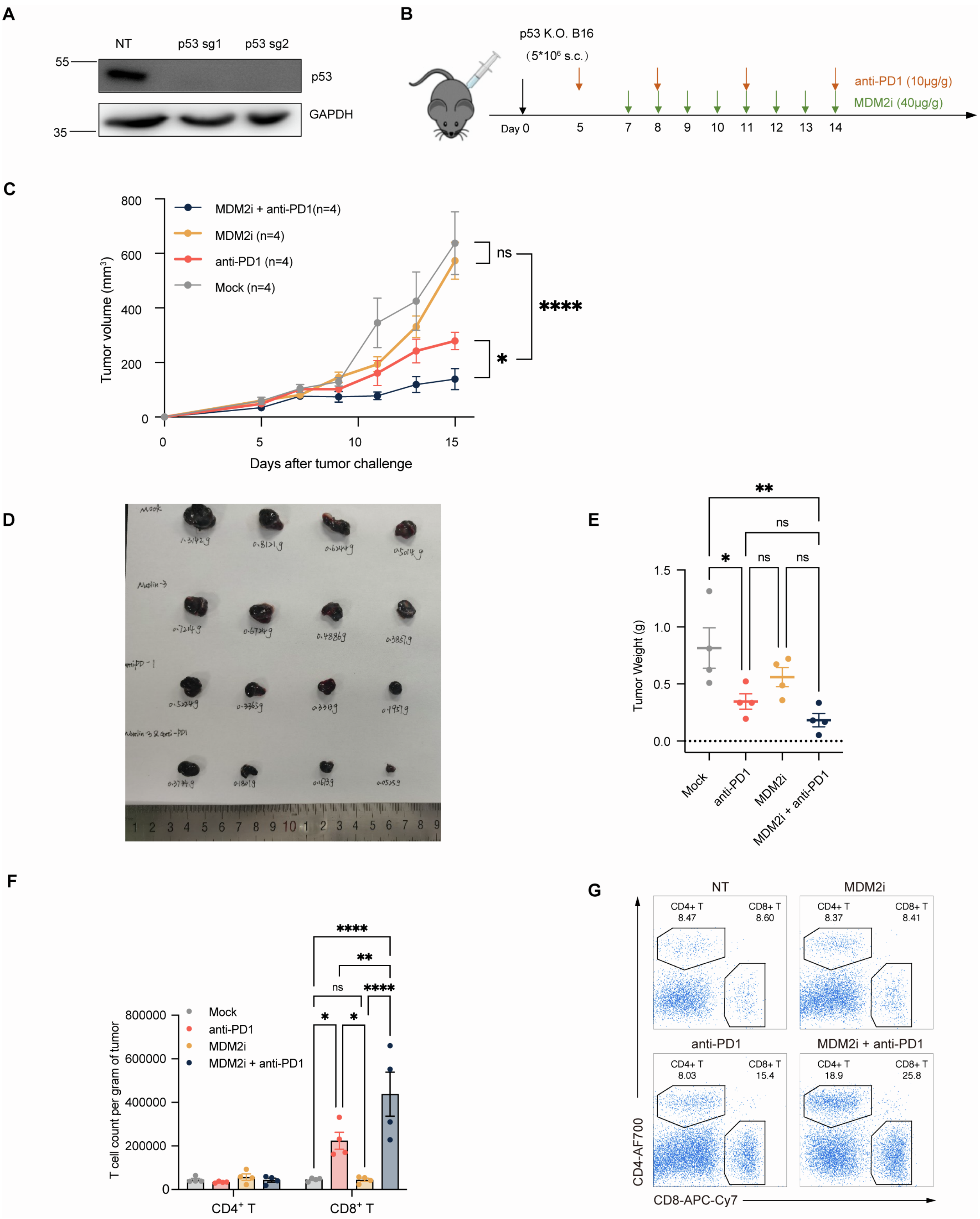

Figure S7. MDM2 deficiency and inhibition promote anti-PD-1 efficacy by enhancing recruitment of T cells within p53-deficient B16 tumors, related to Figure 6.

(A) Knockout efficiency of p53 in B16 was evaluated by western blotting with antibodies against p53.

(B) Experimental design showed C57BL/6 mice were inoculated p53-deficient B16 subcutaneously followed with injecting MDM2i every day and anti-PD-1 once every three days intra-peritoneally.

(C-E) Tumor growth analysis of p53-deficient B16 tumors under different treatment conditions. (**C**) The tumor volume of p53-deficient B16 tumors in different groups were monitored (**D**) then tumors were harvested on day 15 and (**E**) the weights were measured.

(F and G) MDM2 inhibitor Nutlin-3a promoted T cell infiltration with anti-PD-1 in p53-deficient B16. (**F**), Quantification of $CD4^+$ and $CD8^+$ infiltrating T cell counts per gram of tumor. (**G**), The percentage of $CD4^+$ and $CD8^+$ subsets in infiltrated immune cells. Data are represented as mean ± SEM. P values determined by two-way ANOVA with Tukey's correction for multiple comparisons. *P < 0.05, **P < 0.01, and ****P < 0.0001; ns, not significant.
